# Promoting tissue repair with plant-derived compounds: evidence and mechanisms from zebrafish studies

**DOI:** 10.3389/fnut.2026.1794776

**Published:** 2026-06-12

**Authors:** Xiang Meng, Xuting Song, Lixia Zhu, Yiming Wang, Xianghe Meng, Yufang Tang, Kobil A. Bobokalonov, Min He, Mengmeng Sun, Meiying Jin

**Affiliations:** 1 Changchun University of Chinese Medicine, Changchun, China; 2 The Jilin Province School-Enterprise Cooperation Technology Innovation Laboratory of Herbal Efficacy Evaluation Based on Zebrafish Model Organisms, Changchun University of Chinese Medicine, Changchun, China; 3 Jilin Provincial International Cooperation Key Laboratory of Traditional Medicine for Prevention and Treatment of Metabolic Diseases, Changchun University of Chinese Medicine, Changchun, China; 4 Wish Technology, Changchun, China; 5 Institute of Botany, Plant Physiology and Genetics, National Academy of Tajikistan, Dushanbe, Tajikistan; 6 The 3rd Affiliated Hospital of Changchun University of Chinese Medicine, Changchun, China

**Keywords:** phytochemicals, plant-derived compounds, regeneration, tissue repair, zebrafish

## Abstract

The pursuit of strategies to enhance tissue repair is a central challenge in regenerative medicine, where plant-derived compounds offer immense therapeutic potential due to their multi-target, systems-level activities. Validating and mechanistically deciphering these pro-repair effects requires robust, predictive, and ethically tractable *in vivo* models. The zebrafish (*Danio rerio*) has emerged as a preeminent model that powerfully bridges this gap. This review synthesizes the growing body of literature utilizing zebrafish injury models to evaluate the therapeutic potential of herbal extracts and their active constituents. We detail the application of zebrafish models for external wound healing (caudal fin and skin injury), internal tissue repair (bone and vasculature), and chemical-induced organ damage (heart and liver). The collective evidence demonstrates that plant-derived compounds promote repair across these diverse paradigms by modulating evolutionarily conserved mechanisms, including inflammation, oxidative stress, apoptosis, autophagy, and key developmental signaling pathways such as Wnt/*β*-catenin and NF-κB. The unique advantages of the zebrafish—including optical transparency for real-time imaging, genetic tractability, and high physiological conservation with humans—make it an indispensable platform for high-throughput screening and deep mechanistic inquiry. By translating the complex pharmacology of botanicals into actionable insights within a living vertebrate system, zebrafish research accelerates the discovery and validation of the health-promoting effects of natural compounds, firmly establishing their role in tissue regeneration and illuminating a clear path toward their development as next-generation regenerative therapies.

## 1 Introduction

Tissue repair is a cornerstone of organismal survival and a critical determinant of health span, with its dysregulation being a central pathogenic factor in a vast array of debilitating conditions—from chronic wounds and diabetic ulcers to degenerative joint diseases and post-infarction heart failure (1, 84). The successful restoration of tissue integrity hinges on a meticulously coordinated cascade of events, including controlled inflammation, robust cellular proliferation, angiogenesis, and extracellular matrix remodeling (2). However, the efficacy of this cascade is highly context-dependent. It is challenged by the nature of the insult, be it mechanical trauma with its characteristic inflammatory-proliferative response (3, 4), chemical or radiation-induced damage (5), or the progressive micro-injury seen in metabolic and degenerative diseases which impairs fundamental cellular repair mechanisms like autophagy (6, 7). Furthermore, intrinsic factors such as age and systemic metabolic status profoundly influence these repair pathways, often diminishing regenerative capacity (8). This multifaceted complexity underscores the urgent need for therapeutic strategies that can safely and effectively modulate the healing process across diverse injury contexts.

In this therapeutic landscape, plant-derived compounds emerge as a uniquely promising resource. With a history rooted in millennia of traditional medicine, botanicals offer a rich source of multi-target bioactive agents. Compounds from *Salvia miltiorrhiza* and *Panax notoginseng*, for instance, are known to improve microcirculation and resolve stasis (9, 10), while ubiquitous polyphenols exert potent anti-inflammatory effects (11). Critically, the strength of phytochemicals lies in their inherent polypharmacology—their ability to simultaneously engage multiple targets within interconnected signaling networks, such as those governing oxidative stress, immune response, and cell survival (12). This systems-level action allows them to promote regeneration by coordinately activating repair signals, suppressing maladaptive inflammation and apoptosis, and enhancing endogenous antioxidant defenses. This multi-pronged approach can potentially overcome limitations of single-target therapies, including side effects and resistance, while often maintaining a favorable safety and cost-effectiveness profile (13, 14). The current frontier of research thus focuses on rigorously validating these pro-repair effects and isolating the novel bioactive principles responsible.

To bridge the gap between traditional use and modern mechanistic understanding, robust and predictive *in vivo* models are essential. The zebrafish (*Danio rerio*) has ascended as a preeminent model organism for regenerative medicine and drug discovery. Its unparalleled value stems from a convergent set of advantages: a profound natural capacity to regenerate a wide array of tissues, including fins, heart, spinal cord, and kidney, replicating complex repair processes (15, 16); optical transparency during early development, permitting real-time, high-resolution imaging of cellular dynamics during wound healing and regeneration (17, 83); and a high degree of genetic and physiological conservation with humans, with approximately 87% of human disease-related genes having a functional zebrafish ortholog (18, 87). This combination makes the zebrafish an ideal *in vivo* platform for high-throughput screening and functional validation of natural products. It enables researchers not only to observe holistic therapeutic outcomes—such as accelerated fin regrowth or reduced scar formation—but also to dissect the underlying mechanisms in a living, vertebrate system (18).

**Figure 1:**
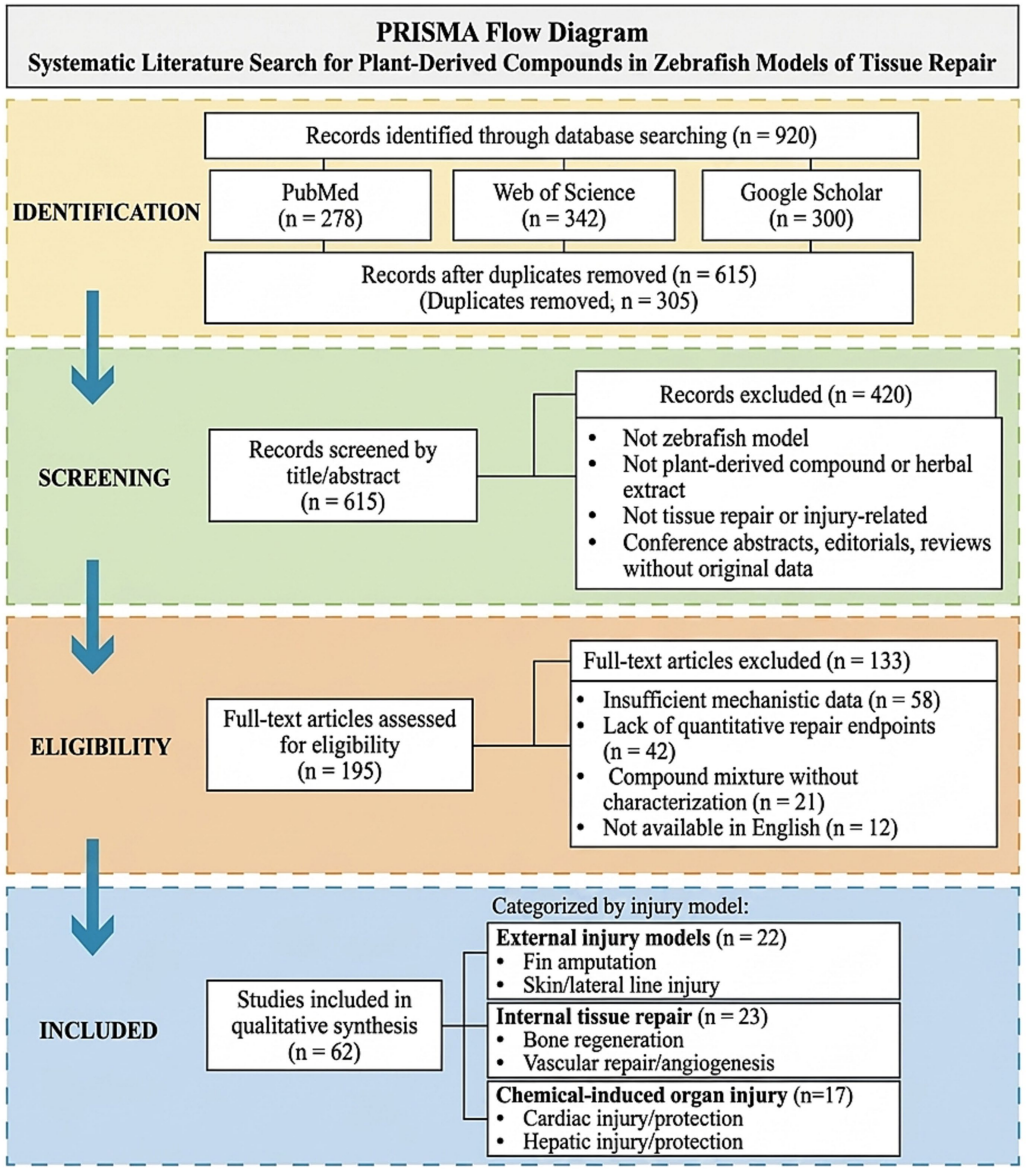
PRISMA-style flow diagram of the literature search and selection process. Databases were searched from 2005 to December 2025 using the following keyword combinations: (*zebrafish OR Danio rerio*) AN*D* (*plant extract OR herbal OR phytochemical OR natural product*) *AND (tissue repair OR wound healing OR regeneration OR bone repair OR angiogenesis OR hepatoprotective OR cardioprotective)*.

This review, therefore, synthesizes literature from the past decades (Web of Science, PubMed, and Google Scholar) to critically assess the role of zebrafish models in evaluating the health-promoting effects of plant-derived compounds on tissue repair (Figure 1). We provide a systematic overview of established zebrafish injury models relevant to both external and internal tissue damage, detail the key phenotypic and molecular readouts used for assessment, and compile the evidence for the reparative bioactivities of numerous herbal extracts and their active constituents. By integrating findings across different injury paradigms—from caudal fin amputation to chemical-induced organ damage—this review aims to clarify how zebrafish research accelerates the discovery and mechanistic elucidation of plant-based reparative agents, offering a robust scientific rationale for their further development into novel regenerative therapies.

## 2 Evaluation of plant-derived extracts and active compounds in zebrafish models of external injury and wound healing

The skin and its appendages serve as the primary barrier against the external environment, making injuries to these structures a major clinical concern. Zebrafish models of external wounding provide unparalleled *in vivo* access to the entire repair continuum, from the initial inflammatory response to the final stages of tissue remodeling. The following sections detail how these models, particularly those involving the tail fin and skin, have been instrumental in quantifying and deconvoluting the therapeutic potential of plant-derived compounds (Table 1). These external injury paradigms primarily evaluate *de novo* tissue regeneration, where the endpoint is the formation of new functional tissue through endogenous morphogenetic programs.

**Table 1:** Therapeutic potential of herbal medicine for external injury and tissue repair using a zebrafish model.

Model category	Herb	Component	Model	Phase of action	Changes in indicators	References
Fin regeneration	*Eckl onia cava* (E. cava)	Fucoidan extraction	Tail fin amputation (larvae) and LPS exposure (embryos)	Inhibit ROS and NO generation; protect against cardiotoxicity	↓: ROS, NO, heart rate, yolk sac edema size	(91)
	*Antrodia cinnamomea*	ethanol extraction waste (ACEW)	Caudal fin amputation (adult) and oxazolone-induced enteritis (adult)	Anti-inflammation; promote tissue regeneration; improve stress tolerance	↓: *tnfa*, *il1b*, *il6*, *il10*; ↑: *il4*/*il13a*, fin regeneration area, survival under thermal stress	(92)
	*Piper Sarmento sum* (Roxb.)	aqueous extraction	Caudal fin amputation (adult)	Sex-dependent modulation of regeneration	Male zebrafish showed poor regenerative capacities of pectoral fins when compared to female zebrafish.	(35)
	*Arthrospira*	Spirulina maxima-based pectin (SmP)	Tail fin amputation (larvae) and laser-induced dermal wound (adult)	Promote fibroblast proliferation/migration; enhance re-epithelialization and tissue remodeling; upregulate wound healing-related genes	↑: HDF proliferation, fin regeneration area, wound closure %, pigment restoration; ↑: *tgfb1*, *mmp9*, *mmp13*, *timp2b*, *tnfa*, *il1b* (early phase)	(93)
	*Propolis* (Colla Apis)	ethanol extraction	Caudal fin amputation (adult hyperglycemic model)	Antioxidant; upregulate Hedgehog, BMP, and Wnt signaling	↓: Hyperglycemia-impaired regeneration; ↑: *shha*, *igf2a*, *bmp2b*, *col1a2*, fin regeneration %	(34)
	*Betula platyphylla* Suk.	Betulin	Tail fin amputation (embryos)	Inhibit ROS/MAPK/NF-κB pathway; reduce apoptosis	↓: ROS, melanin aggregation, *casp3a*; ↑: Fin regeneration area	(31)
Fin regeneration	*Siparuna guianensis*	Methanol extraction (ME), 70% ethyl alcohol extraction (HE), and distilled water extraction (AE)	Caudal fin amputation (adult)	Anti-inflammatory; no effect on fin regeneration or antioxidant enzyme activity	↓: Inflammation; ↔: Fin regeneration, SOD, CAT	(94)
	*Cannabis*	Cannabidiol (CBD) ethanol extracts	Tail fin amputation (embryos)	Modulate IL-1β/Caspase-3/PARP pathway; anti-inflammatory	↑: Fin regeneration; ↓: *il1b*, *casp3a*, *parp*	(95)
	Lemongrass (*Cymbopogon citratus*)	*Cymbopogon citratus* essential oil	Tail fin amputation (embryos)	Anti-inflammatory; delayed fin regeneration	↓: Inflammation; ↑: Fin healing time (slowed regeneration)	(96)
	*Urtica cannabina L.* (UL)	--------	Caudal fin amputation (adult)	Anti-inflammatory; promote fin regeneration	↑: Fin regeneration; ↓: *tnfa*, *il8*, *il6*	(97)
	*Rehmannia glutinosa*	Radix Rehmanniae Praeparata (RRP)	Tail fin amputation (embryos; *Tg(cmv: GFP-LC3)*)	Activate AHR signaling and autophagy; promote blastema cell proliferation	↑: Fin regeneration, autophagic flux, blastema proliferation	(32)
Fin regeneration	*Achillea alpina* L.	*Achillea alpina* L. essential oil (AHO)	Tail fin amputation [*Tg(MPX:eGFP)*; *Tg(LC3: eGFP)*]	Anti-inflammatory; inhibit neutrophil aggregation; modulate autophagy	↓: Neutrophil recruitment, *tnfa*, *il1b*, *il6*; ↑: Fin regeneration (via autophagy modulation)	(33)
	*Panax ginseng*	Ginsenosides Rg1	Tail fin amputation (embryos; *Tg(mpx: GFP/mpeg1: mCherry)* and *gr<sup>s357</sup>* mutant)	Selective GR agonist; inhibit neutrophil migration via GR transrepression without impairing tissue regeneration	↓: Neutrophil migration (*gr*-dependent); ↔: Fin regeneration	(29)
	*Sophora flavescens* Ait. (SFA)	Oxymatrine (OMT)	Tail fin amputation (embryos; *Tg(MPX:eGFP)*)	Inhibit neutrophil migration; modulate MAPK signaling	↓: Neutrophil recruitment; ↑: *jnk*, *casp3*, *mapk14a*, *mapkapk2a*, *map2k1*	(30)
	--------	Silver nanoparticles (AgNPs)	Caudal fin amputation (embryos; *Tg(lyz: DsRed)* and Tg(mpeg1: loxp-DsRed-loxp-EGFP))	Inhibit cell proliferation in regenerative blastema; decline amputation-induced ROS; promote neutrophil recruitment	↓: Fin regeneration, cell proliferation, ROS; ↑: Neutrophil recruitment (early phase)	(98)
Fin regeneration	--------	Aristolochic Acid	Tail fin amputation (embryos; *Tg(VEGFR2: GFP)*)	Anti-angiogenic; induce developmental and cardiovascular toxicity	↓: Fin regeneration, ISV/DLAV length and diameter, heart rate, hatching rate; ↑: Pericardial edema, yolk sac edema, mortality	(99)
	*Lithospermum erythrorhizon*	Shi Konin	Tail fin amputation (embryos; *Tg(lyz: DsRed)*, *Tg(coro1a: GFP)*)	Upregulate oxidative stress and AMPK signaling; inhibit fin regeneration	↓: Fin regeneration; ↑: ROS, AMPK pathway activation	(100)
	*Hypericum lanceolatum* Lam.	aqueous extraction	Tail fin amputation (embryos; *Tg(GFAP: GFP)*)	Improve antioxidant response; enhance immune cell infiltration at injury site	↑: Fin regeneration, antioxidant activity, immune cell recruitment	(101)
	*Pouteria lucuma*	Fatty acids (FAs)	Tail fin amputation [*Tg(fli1: EGFP)*]	Promote fin regeneration (linoleic and oleic acids as major active FAs); no significant antibacterial or antifungal activity	↑: Fin regeneration; ↔: Antibacterial/antifungal activity	(102)
	Comfrey (*Symphytum officinale L.*)	ethanol extraction	UV-induced fin injury (embryos)	Upregulate *bcl2*; reduce ROS; protect against UV-induced apoptosis	↓: ROS, apoptosis; ↑: *bcl2*	(103)
Skin regeneration	*Elaeis guineensis* Jacq.	extract of OPL (OPL-FEE) and (OPL-FEE-NE)	Skin biopsy punch injury (adult)	Anti-inflammatory; antioxidant; promote wound closure with minimal scarring	↑: *mmp9*, *mmp13*, *cat*; ↓: Inflammation, scar formation	(38)
	*Azadirachta indica*	--------	Lateral line injury (adult)	Promote wound contraction; stimulate fibroblast proliferation	↑: Fibroblast proliferation	(39)

These abstract use wild-type and transgenic zebrafish as experimental models to provide information on the regulatory effects of natural product extracts or monomeric components on fin amputation, skin wound, and lateral line injury repair. “↑” indicates increase, upregulation, or promotion; “↓” indicates decrease, downregulation, or inhibition; “↔” indicates no significant change.

### 2.1 The zebrafish tail fin injury model: a dynamic platform for deciphering herbal pro-repair mechanisms

The amputated zebrafish tail fin is a preeminent *in vivo* model for elucidating the coordinated multi-phase process of wound healing and regeneration (19). Induced via precise transection, this model provides a temporally resolved view of repair, making it an ideal system for screening plant-derived compounds and dissecting their mechanisms of action. Regeneration progresses through four consecutive, overlapping stages: an initial inflammatory phase marked by wound epithelium formation and the release of mediators like IL-1*β* and TNF-*α* (20); a blastema formation stage governed by pathways such as Wnt/β-catenin and FGF; a regenerative outgrowth phase where reactive oxygen species (ROS) and BMP signaling guide proliferation and differentiation (21); and a final remodeling stage involving vascular and nervous repair, with metrics like vascular density indicating healing quality (22–25).

The true power of this model lies in its ability to link specific phenotypic readouts to molecular mechanisms, a feature extensively leveraged to validate herbal pro-repair effects. Research has demonstrated that many compounds accelerate healing by strategically intervening at different stages of this cascade. For instance, early inflammatory responses are a common target, with compounds like cannabidiol (CBD) and lemongrass essential oil shown to dampen inflammation and promote tissue repair by modulating the IL-1β/Caspase-3/PARP axis (26, 27). This anti-inflammatory capacity is further highlighted by stinging nettle (*Urtica dioica* L.) extract, which significantly reduces levels of key cytokines including TNF-*α*, IL-8, and IL-6 (28). The advent of transgenic zebrafish lines has been pivotal for pinpointing such actions, enabling live imaging that reveals how ginsenoside Rg1 and oxymatrine specifically inhibit neutrophil migration to the wound site (29, 30).

Beyond inflammation, the model reveals how compounds interact with fundamental regenerative signals. The regeneration-associated burst of ROS, for example, is a key therapeutic node. Betulin, a birch-derived compound, promoted fin regeneration and was associated with reduced expression of ROS/MAPK/NF-κB pathway components and downregulation of pro-apoptotic Caspase-3, suggesting a multi-level anti-inflammatory and anti-apoptotic action (31). Furthermore, critical intracellular processes like autophagy have been implicated through the use of specialized transgenic lines. Studies utilizing Tg(cmv: GFP-LC3) zebrafish indicate that *Rehmannia glutinosa* praeparata and *Achillea alpina* essential oil promote fin regeneration through mechanisms that involve autophagy activation and AHR signaling, as evidenced by increased autophagic flux, enhanced blastema cell proliferation, and upregulation of pro-regenerative genes (32, 33). Pharmacological inhibition of AHR partially attenuated these effects, providing functional support for this pathway’s involvement.

The utility of the tail fin model also extends to modeling complex, disease-impaired healing. In hyperglycemic zebrafish, which mimic the compromised repair seen in diabetes, propolis extract rescued fin regeneration and was accompanied by the upregulation of repair-associated genes including *shha*, *igf2a*, *bmp2b*, and *col1a2* (34). Moreover, Zainol Abidin et al. (35) demonstrated that male zebrafish exhibited significantly slower caudal fin regeneration than females after amputation, and aqueous extracts of *Piper sarmentosum* differentially improved regrowth in males while showing limited additional benefit in females. This sexual dimorphism may arise from sex hormone-dependent differences in inflammatory responses, cellular proliferation rates, and oxidative stress handling, underscoring the necessity of considering sex as a biological variable in regenerative studies.

It is equally instructive to consider compounds that impair fin regeneration, as they illuminate the fragile boundary between therapeutic efficacy and toxicity. Silver nanoparticles (AgNP), widely used in wound dressings, significantly inhibited fin regeneration when exposure occurred during the epithelialization and early blastema phases, primarily by suppressing cell proliferation in the regenerative blastema (98). Notably, rather than inducing oxidative damage, AgNP exerted this anti-regenerative effect by decreasing amputation-induced ROS production—likely through enhanced neutrophil recruitment and subsequent myeloperoxidase-mediated H₂O₂ clearance—underscoring that a certain threshold of ROS signaling is indispensable for initiating repair. Similarly, aristolochic acid (AA), a natural compound found in Aristolochia species, attenuated fin regeneration in a dose-dependent manner and caused severe cardiovascular defects, including disrupted intersegmental vessel development and pericardial edema, at concentrations ≥10 μM (99). These negative examples highlight two principles central to the translational development of phytochemicals: first, the existence of narrow therapeutic windows where beneficial and adverse effects are dose-dependent and stage-dependent; and second, the dual role of ROS as both an essential regenerative signal and a potential mediator of toxicity, a concept that must be carefully navigated when designing pro-regenerative therapies. In summary, the zebrafish tail fin model transcends a simple regeneration assay; it is a dynamic, stage-resolved platform that precisely identifies how plant-derived compounds intervene in the repair cascade—from immune modulation and redox balancing to cellular reprogramming and tissue repatterning.

### 2.2 Zebrafish skin injury and healing model

Complementing the tail fin model, zebrafish skin wounding provides a direct and clinically relevant platform for investigating traumatic cutaneous repair and the impact of therapeutic compounds. The skin repair process in zebrafish progresses through defined stages comparable to mammals, including hemostasis, inflammation, re-epithelialization, granulation tissue formation, and remodeling (36). A critical advantage of this system is the distinct healing phenotype observed between developmental stages. Larval zebrafish typically exhibit rapid, scar-free regeneration driven by efficient stem cell activation and a minimal inflammatory response. In contrast, adult zebrafish heal more slowly, with a more pronounced inflammatory phase and a tendency toward fibrotic scarring, thus offering a valuable parallel to imperfect mammalian wound healing (37). This dichotomy makes the model exceptionally useful for probing how interventions can shift the balance from scarring toward regenerative repair.

Research utilizing this model has effectively demonstrated the wound-healing capacities of plant extracts. For instance, a study employing an adult zebrafish biopsy punch model showed that extract from oil palm leaf (*Elaeis guineensis* Jacq.) and its nanoemulsion formulation significantly enhanced healing. These treatments exerted anti-inflammatory and antioxidant effects during the early phase, upregulated key repair genes such as *mmp9*, *mmp13*, and *cat*, and ultimately promoted faster wound closure with minimal scarring (38). This underscores the model’s utility in evaluating multi-parameter outcomes, from molecular signaling to macroscopic tissue closure. Similarly, the model’s adaptability is illustrated in a study using a lateral line injury in juvenile zebrafish, where neem leaf (*Azadirachta indica*) extract was found to accelerate wound area reduction, an effect linked to stimulated fibroblast proliferation at the injury site (39). Together, these examples highlight how zebrafish skin and lateral line injury models, across different life stages and wounding techniques, serve as versatile *in vivo* screens for herbal medicines. Beyond merely assessing regenerative wound closure, these models are uniquely suited for characterizing critical, clinically relevant endpoints such as inflammation resolution, matrix remodeling, and scar mitigation.

## 3 Evaluating plant-derived extracts and active compounds for internal tissue and organ repair in zebrafish

Beyond external wounds, zebrafish provide robust models for investigating repair within deeper tissues and organs, enabling real-time observation of processes that are difficult to track in mammalian systems. The following sections examine their application in bone regeneration and vascular repair, detailing how plant-derived compounds facilitate internal healing (Table 2). Like the surface injury models, these paradigms primarily evaluate regenerative endpoints, such as new bone formation and angiogenesis. However, the initiating insults are broader, encompassing both chemical exposure and physical trauma.

**Table 2:** Therapeutic potential of herbal medicine for internal injury and tissue repair using a zebrafish model.

Model category	Herb	Component	Model	Phase of action	Changes in indicators	References
Promote angiogenesis	*Glycyrrhiza uralensis Fisch.*	Isoliquiritin (ISL)	Skin injury and needle-stick wound [*Tg(fli-1: EGFP)*, *Tg(MPEG: Cherry)*]	Promote macrophage recruitment; pro-angiogenic activity involving VEGF signaling; modulate MMPs and antioxidant enzymes	↑: Macrophage recruitment, wound healing, *vegfa*, *kdr* (flk-1), tie-1, *tgfb*, *mmp9*, *mmp13*, *sod1*	(89)
	*Uncaria gambir Roxb*	Catechu	Wild-type AB and *Tg(fli1: EGFP)*	Induce angiogenesis; activate FGF/FGFR, NF-κB, and STAT3 signaling; upregulate vimentin	↑: SIV sprout number and length, *il8* (*cxcl8a*), *fgfr2*, *fgfr3*, *nfkb*, *stat3*	(104)
	*Astragali Radix*	Astragali Radix (ARP)	VEGFR tyrosine kinase inhibitor II (VRI)-induced blood vessel loss [*Tg(fli1a: EGFP)*]	Reverse VRI-induced downregulation of VEGFR genes; protect against chemical-induced vascular regression	↑: ISV length recovery, endothelial cell number, *kdrl*, *kdr* (flk-1), *flt1* (*vegfr1*) mRNA	(105)
	*Angelica sinensis* (AS)	Angelica sinensis extract	*Tg(fli1: EGFP)* embryos	Promote angiogenesis; activate p38 and JNK1/2 phosphorylation	↑: *vegfa* mRNA, SIV sprouting, p38/JNK phosphorylation	(106)
	*Rehmannia glutinosa* (RR)	aqueous crude extract	*Tg(fli1: EGFP)* embryos	Enhance VEGF expression; promote angiogenesis	↑: *vegfa*, SIV capillary sprout formation	(60)
Promote angiogenesis	*Leonurus japonicus Houtt.*	Motherwort total alkaloids	Sunitinib-induced ISV loss [*Tg(flk1: EGFP)* embryos]	Promote angiogenesis	↑: ISV recovery (dose-dependent)	(107)
	*Carthami flos*	*Carthami flos* (CF) whole extract	*Tg(fli1: EGFP) embryos (SIV angiogenesis assay)*	Promote proliferation, migration and tube formation of endothelial cells; upregulate multiple angiogenesis-related genes	↑: HMEC-1 cell proliferation, migration, tube formation; ↑: SIV sprout number; ↑: *igf1*, *ctgf*, *nrp2*, *vegfr3*, *hif1a*, *mmp2*, *mmp9*, *timp2*, *plg*, *plau*, *itgav*, *itgb3*, *ctnnb1* (β-catenin), *pecam1*, *angpt1*, *tie2*, *pdgfrb*, *cdh5*, *s1pr1*, *fgf2*, *shh*, *tgfrb1*, *efnb2* (Ephrin B2)	(108)
	Tongnao Decoction (TND)	--------	VRI-induced impairment of ISVs, SIVs, and CtAs [*Tg(fli-1: EGFP)* embryos]	Activate VEGFR-2, PI3K/Akt, and Raf/MEK1/2/ERK1/2 signaling pathways (validated by specific kinase inhibitors)	↑: HUVEC proliferation, migration, tube formation; ↑: ISV, SIV, and CtA recovery in VRI-damaged embryos; pro-angiogenic effect blocked by VEGFR2i, PI3Ki, Akti, Rafi, MEK1/2i, ERK1/2i	(61)
	--------	Resveratrol	Tg (flil: EGFP) zebrafish staining with Carboxy-H2DCFDA probe.	Inhibit ROS production; preserve mitochondrial membrane potential; prevent ZnO NP-induced apoptosis and necrosis	↓: ROS, mitochondrial dysfunction, apoptosis/necrosis; ↑: Vascular structural integrity	(58)
Promote angiogenesis	Sanwujiao granule	--------	VEGFR inhibitor (VRI)-induced vascular deficiency [*Tg(kdrl:eGFP)* zebrafish larvae]	Promote angiogenesis; upregulate IGF-1 and Notch-1 signaling	↑: Total BV length, intact ISV number; ↑: *igf1*, *notch1* mRNA	(57)
	*Corydalis decumbens* (Thunb.) Pers.	sinometumine E(SE)	PTK787-, MPTP-, and atorvastatin-induced vascular injury models	Regulate HIF-1/VEGF signaling pathway; promote angiogenesis; block VEGFR autophosphorylation; inhibit excessive endothelial cell proliferation	↑: Angiogenesis (HIF-1/VEGF pathway); ↓: VEGFR autophosphorylation, pathological endothelial proliferation	(55)
	*Trichosanthis fruit* (TF)	Paeonol (Pae), diosmetin-7-O-β-D-glucopyranoside (diosmetin-7-O-glucoside),5-hydroxymethylfurfural (5-HMF)	Arachidonic acid (AA)-induced thrombosis and inflammation [*Tg(coo1a: EGFP)*, *Tg(CD41: eGFP)*]	Anti-thrombotic; anti-inflammatory; normalize coagulation and prostaglandin synthesis gene expression	↑: Thrombosis resolution, inflammatory response improvement; ↓: *f2* (thrombin), *fga*, *fgb*, *vwf*, *ptgs1*, *tbxas1* expression	(109)
Bone regeneration	Curcuma-bromelain-papain-pepper herbal preparation (CHP)	--------	Prednisolone-induced osteoporosis (scales) (adult zebrafish)	Counteract glucocorticoid-induced osteoporosis; maintain osteoblast and osteocyte metabolic activity	↑: ALP activity, osteoblast viability; ↓: Osteoporotic phenotype	(45)
	*Panax ginseng*	Ginsenoside Rg1	Dexamethasone-induced vertebral impairment (zebrafish larvae)	Activate GPER; promote PI3K/AKT phosphorylation; enhance osteoblast differentiation and mineralization	↑: Bone mineralization (alizarin red staining), *runx2a*, *bglap* (osteocalcin), *alp* mRNA; ↓: Dexamethasone-induced bone loss	(46)
	Colla Carapacis et Plastri, CCP	--------	Wild-type zebrafish embryos	Upregulate ossification-related genes and proteins; promote osteogenesis	↑: *runx2a*, *bglap* (osteocalcin), *spp1* (osteopontin) mRNA/protein; ↑: Osteogenesis	(110)
	*Amomum villosu*m Lour. and *Morus alba* L.	Isoquercitrin (IQ)	AAPH-induced oxidative injury (bone dysplasia model) (zebrafish)	Activate Keap1-Nrf2-ARE antioxidant pathway; inhibit Caspase-3-mediated apoptosis	↑: Bone development, *nrf2*, ho-1, *nqo1*; ↓: *keap1*, *casp3a*, ROS, oxidative damage	(20)
	*Safflower*	HSYA	TAA-induced bone mass reduction (zebrafish larvae; Wild-type AB)	Anti-inflammatory; antioxidant; modulate RANKL/OPG ratio	↑: Bone mineralization; ↓: *il6*, *il1b*, *tnfa*, oxidative stress markers; ↓: *rankl*/*opg* ratio	(111)
Liver protection	*Radix Polygoni Multiflori Preparata* (RPMP)	emodin	Egg yolk powder-induced NAFLD (zebrafish larvae Wild-type AB)	Reduce hepatic lipogenesis; activate PI3K/AKT2/AMPKα and PPAR*α* signaling	↓: Hepatic lipid accumulation; ↑: *pik3ca*, *akt2*, *ampkα*, *pparα*, *cpt1a*, *acox1*	(89)
	Lian-Mei-Yin (LMY)	--------	Diet-induced NAFLD (zebrafish larvae; Wild-type AB)	Inhibit Yap1-mediated Foxm1 activation; reduce lipogenesis and hepatic lipid accumulation	↓: Hepatic steatosis, *yap1*, *foxm1*, lipogenesis	(112)
Liver protection	*Gastrodia elata* Blume	Gastrodin (GAS)	High-fat diet (HCD)-induced non-alcoholic fatty liver disease (NAFLD) (zebrafish larvae; Wild-type AB)	Reduce oxidative stress; inhibit lipogenesis and inflammation; activate Nrf2 antioxidant pathway	↓: Mortality, body weight, TG, TC, ROS, MDA, *srebp1*, *fasn*, *tnfa*, *il6*, *il1b*, *tgfb*, *keap1*	(113)
	Yihe-Tang	--------	Constant dark-induced NAFLD (zebrafish; Wild-type AB)	Promote lipolysis; inhibit fatty acid synthesis; restore gut microbiota composition	↑: *cpt1*, *acadm*; ↓: *fasn*	(114)
Liver protection	*Lycii Fructus*	LFP-a1	TAA-induced liver injury (zebrafish larvae; Wild-type AB)	Antioxidant; anti-apoptotic; preserve liver integrity	↑: CAT, GSH-Px, GSH, T-AOC, *bcl2*; ↓: ALT, AST, ROS, NO, MDA, *bax*, *casp3a*, *c-myc*	(115)
	*Chrysanthemum morifolium Ramat*	water extract from chrysanthemum flowers (WEFC)	TAA-induced fatty liver disease [*Tg(fabp10a: DsRed)*]	Hepatoprotective; activate AKT1-mediated ADIPOQ/PPARs lipid metabolism pathway	↑: Liver area, fluorescence intensity, *adipoq*, *akt1*, *ppara*, *ppary*; ↓: Yolk sac area, hepatic steatosis, TG, TC, *sreb1*, *fasn*, *mttp*	(82)

These abstract use wild-type and transgenic zebrafish as experimental models to provide information on the regulatory effects of natural product extracts or monomeric components on angiogenesis, bone regeneration, vascular protection, and metabolic liver protection. “↑” indicates increase, upregulation, or promotion; “↓” indicates decrease, downregulation, or inhibition; “↔” indicates no significant change.

### 3.1 Zebrafish models in skeletal repair and the therapeutic actions of phytochemicals

The zebrafish has emerged as a highly tractable model for skeletal biology, providing significant insights into human bone disorders such as osteoporosis and enabling the evaluation of natural products for bone regeneration. This utility stems from the remarkable conservation of bone development, composition, and remodeling processes between teleost fish and humans (40, 41). The bone healing process in zebrafish recapitulates key mammalian stages—inflammation, cartilaginous callus or direct bone matrix formation, mineralization, and remodeling—which depend on a delicate balance between osteoblastic bone formation and osteoclastic resorption. Disruptions in this homeostasis underlie pathologies like osteoporosis and osteoarthritis (42). Leveraging various injury models, including glucocorticoid-induced osteoporosis, spinal lesions, and fin bone fractures, researchers can probe these dynamics using techniques from mechanical and chemical induction to genetic manipulation (43, 44). Key molecular players such as BMP, Wnt, Runx2, RANKL, and OPG are conserved, allowing assessment through parameters like bone density, length, histology, and gene expression (Table 2).

This robust platform has been effectively used to demonstrate the osteogenic and osteoprotective properties of numerous plant-derived compounds. For instance, a herbal preparation of curcuma, bromelain, papain, and pepper (CHP) countered glucocorticoid-induced osteoporotic phenotypes in adult zebrafish scales. Its active component, curcumin, helped maintain osteoblast and osteocyte metabolic activity, restoring the balance of key markers like alkaline phosphatase (ALP) and tartrate-resistant acid phosphatase (TRAP) to promote repair (45). Similarly, the ginsenoside Rg1 was shown to alleviate dexamethasone-induced vertebral impairment in larvae, enhancing bone mineralization and upregulating the osteogenic master gene runx2a. This anabolic effect appears mediated through the G protein-coupled estrogen receptor (GPER) and the subsequent activation of the PI3K/AKT signaling pathway (46). *Notably, the mechanism engaged by Rg1 in bone differs from that observed in the fin amputation model (Section 2.1), where the same compound acts as a selective glucocorticoid receptor (GR) agonist to suppress neutrophil migration without impairing regenerative outgrowth (29). This dichotomy illustrates how a single phytochemical can engage distinct signaling modules in a tissue and injury-specific manner. Other compounds target the inflammatory and oxidative stressors that compromise bone integrity. Hydroxysafflor yellow A (HSYA) from safflower enhanced mineralization in larvae, reduced levels of pro-inflammatory cytokines (IL-6, IL-1β, TNF-*α*), lowered oxidative stress markers, and modulated the RANKL/OPG ratio, suggesting a coordinated anti-inflammatory and bone-protective action (47). Furthermore, the flavonol isoquercitrin (IQ) accelerated osteogenesis, as evidenced by enhanced calcein staining, and this effect was linked to activation of the Keap1-Nrf2-ARE antioxidant pathway and inhibition of Caspase-3-mediated apoptosis. Nrf2 knockdown partially abolished the osteoprotective effects, providing functional evidence for this mechanism (48). Collectively, these studies underscore how the zebrafish skeletal model translates traditional herbal knowledge into validated, mechanistically defined pro-osteogenic strategies.

### 3.2 The zebrafish vascular system: a window into herbal angiogenic and vaso-protective mechanisms

The formation of new blood vessels (angiogenesis) and the protection of existing vasculature are fundamental to the healing of most tissues, as they restore oxygen and nutrient supply while facilitating the removal of waste (49). The zebrafish model is exceptionally powerful for vascular studies, capitalizing on the optical transparency of its embryos and larvae which allows for direct, real-time visualization of vascular architecture and blood flow in living organisms. Coupled with a high degree of conservation in vascular development and signaling pathways with humans, this makes zebrafish an ideal system for evaluating the angiogenic and vasoprotective effects of natural products (50). Consequently, zebrafish are widely used to model a spectrum of vascular disorders, including impaired angiogenesis, thrombosis, and the vascular complications of diabetes, with analysis spanning morphology, function, and molecular markers of key pathways involving VEGF, HIF-1α, and inflammatory cytokines (Table 2).

The multi-faceted activities of plant-derived compounds—encompassing antioxidant, anti-inflammatory, and anti-apoptotic properties—position them as promising agents for vascular repair (51–53). Research in zebrafish has vividly illustrated these potentials across different injury contexts. In models of diabetic vasculopathy, extracts such as Catechu and Brazilian green propolis water extract (WEP) have been shown to counteract hyperglycemia-induced damage. Catechu improved intestinal vascular sprouting (54), while WEP protected the delicate vasculature of the central nervous system and retina from glucose-induced malformations (34). For ischemic injuries mimicking stroke, sophisticated chemical or genetic vascular injury models have revealed detailed mechanisms. The alkaloid sinometumine E from *Corydalis decumbens* exhibited a multi-target action, regulating the HIF-1/VEGF pathway to promote reparative angiogenesis while inhibiting harmful endothelial proliferation (55). Similarly, Sanwujiao granules and *Astragalus* polysaccharide fraction P4 promoted cerebral vessel growth and countered vascular regression by upregulating pro-angiogenic factors like IGF-1 and VEGFRs (56, 57).

Beyond chemical induction, mechanical injury models offer insights into trauma repair. Using a needle puncture model in transgenic larvae, isoliquiritin (ISL) from licorice demonstrated a dual function: it robustly promoted new blood vessel growth while simultaneously enhancing the recruitment of macrophages to the wound site, highlighting a coordinated immuno-angiogenic response crucial for repair (88, 89). Furthermore, the model is adept at assessing protection against toxic insults, as shown by resveratrol’s ability to mitigate nanoparticle-induced oxidative stress and vascular degeneration (58). The utility of zebrafish for straightforward angiogenic screening is also well-established, with classic compounds and formulas like extracts from *Rehmannia glutinosa*, Angelica sinensis, and Erxian Decoction consistently promoting subintestinal vessel growth through the upregulation of VEGF signaling and related kinases (59–62). Collectively, these studies underscore how the zebrafish vascular model serves as a versatile and revealing platform, not only for identifying herbal compounds with therapeutic potential but for delineating the precise cellular and molecular interactions through which they stabilize, repair, and regenerate the circulatory system.

## 4 Protective effects of plant-derived extracts and active compounds against chemical-induced organ injury in zebrafish

Unlike the regenerative paradigms discussed in Sections 2 and 3, which primarily involve physical injury, this section evaluates the protective efficacy of plant-derived compounds against chemical-induced organ damage. Chemical insults, ranging from environmental toxins to pharmaceutical side effects, pose a significant threat to organ integrity. Consequently, the primary endpoint in these studies is not the promotion of *de novo* tissue growth, but rather cytoprotection. The focus shifts to preserving cell viability and organ function by attenuating oxidative stress, inflammation, and apoptosis. Given its capacity for high-throughput screening and direct *in vivo* observation of organ-level toxicity, the zebrafish provides a robust platform for evaluating this protective potential. The following sections detail its application in two primary targets of chemical injury, the heart and the liver (Table 3).

**Table 3:** Therapeutic potential of herbal medicine for chemical-induced organ injury and healing using a zebrafish model.

Model category	Herb	Component	Model	Phase of action	Changes in indicators	References
Heart protection	*Artemisia annua* L.	Artesunate (ART)	Verapamil-induced heart failure (zebrafish larvae; Wild-type AB)	Cardioprotective at low doses; modulate Wnt/β-catenin signaling (*fzd7a*); reverse mitochondrial dysfunction (*ndufa8*); cardiotoxic at high doses	At 1/2 LOAEL: ↑cardiac function, cardiac output, blood flow; restoring normal expression of *fzd7a*, *ndufa8*, *cdk5rap1*, *ptges*. At higher doses: ↓ heart rate, ↑ pericardial edema, yolk sac edema	(69)
	Chinese water chestnuts	Lactate	Zebrafish AHF model	Anti-inflammatory; inhibit cardiac hypertrophy; reduce MAPK signaling activity; regulate stem cell behavior through fine-tuning the inflammatory response	↓: Cardiac hypertrophy, inflammation, MAPK pathway activation	(67)
	Panacis Quinquefolii Radix (PQR)	Ginsenoside	Verapamil hydrochloride-induced heart failure (48 hpf zebrafish larvae)	Increase ventricular myocyte viability via AMPK pathway activation; anti-apoptotic; promote energy production	↓: Ventricular myocyte apoptosis; ↑: AMPK metabolic pathway activity, myocardial viability	(68)
Liver protection	--------	Decabromodiphenyl ethane (DBDPE)	Partial hepatectomy (~80% ventral lobe) in adult zebrafish [*Tg(fabp10a: DsRed; ela3l: EGFP)*] and Wild-type AB	Obstruct liver recovery; environmental pollutant-induced hepatic regenerative impairment	↓: Liver regeneration, regrowth of excised hepatic tissue	(116)
Liver protection	*Loranthus tanakae* Franch. & Sav.	Rhamnetin 3-O-α-rhamnoside (ARR)	TAA-induced acute liver injury (ALI) [*Tg (−1.7apo2: GFP)* larvae]	Antioxidant; inhibit IKKβ/NF-κB signaling pathway; reduce inflammation	↓: Liver damage, oxidative stress, release of intracellular inflammatory factors; ↓: IKKβ/NF-κB pathway activation	(76)
	*Safflower*	HSYA	TAA-induced liver injury (zebrafish larvae; Wild-type AB)	Antioxidant; anti-inflammatory; modulate SIRT1/HMGB1/TLR4/MyD88/NF-κB signaling	↓: Liver damage, oxidative stress, *sirt1*, *hmgb1*, *tlr4*, *myd88*, *nfkb*; ↑: Antioxidant defense	(47)
	*Salvia plebeia* R. Br. (Labiatae)	ethanol extracts of *S. plebeia* (SPEE)	TAA-induced liver injury (adult zebrafish; Wild-type AB)	Anti-inflammatory; anti-apoptotic; downregulate pro-inflammatory cytokines	↓: *il1b*, *tnfa*, hepatocyte apoptosis; ↑: *tgfb*	(74)
	--------	Chlorogenic acid (CGA)	Thioacetamide (TAA) -induced hepatotoxicity [*Tg(fabp10a: DsRed)* larvae]	Anti-apoptotic; pro-proliferative; antioxidant; activate Wnt signaling pathway	↓: Hepatocyte apoptosis, oxidative stress; ↑: Hepatocyte proliferation, liver development; Wnt signaling activation	(75)
Liver protection	*Curcuma phaeocaulis* Val. (PEZ)	Methanol extraction	TAA-induced liver injury (zebrafish larvae; Wild-type AB)	Inhibit TLR4/MyD88/NF-κB signaling pathway at both mRNA and protein levels	↓: *tlr4*, *myd88*, *nfkb*; ↓: TAA-induced liver injury	(73)
	--------	Baicalein	TAA-induced liver injury [zebrafish larvae; Wild-type AB, *Tg(fabp10a: DsRed)*, *Tg(lfabp10a: eGFP)*]	Hepatoprotective via MAPK signaling pathway; modulate inflammatory cytokine expression	↓: *ifng*, *il8* (*cxcl8a*); ↑: *tnfa*, *il1b*	(78)
	Livogrit	--------	TAA-induced hepatotoxicity (zebrafish; Wild-type AB)	Ameliorate alcoholic steatosis; modulate lipogenesis, inflammation, and autophagy via SREBP1c, NF-κB, and LC3A pathways	↓: *srebp1c*, *fas*, *plin2*, *tnfa*, *nfkb*; ↑: *lc3a* (autophagy)	(117)
	*Carthamus tinctorius* L(Safflower)	Hydroxysafflor yellow A (HSYA)	Acetaminophen (APAP)-induced liver toxicity [adult zebrafish; Wild-type AB, *Tg(fabp10a: DsRed; ela3l: EGFP)*]	Accelerate APAP excretion; mitigate APAP-induced hepatotoxicity	↓: APAP toxicity, liver developmental impairment; ↑: APAP clearance	(118)
Liver protection	*Terminalia chebula* Retz	chebulinic acid (CA)	Acetaminophen (APAP)-induced liver injury [*Tg(fabp10a: DsRed; ela3l: EGFP)* larvae]	Activate MAPK/Nrf2 signaling; block ROS production; reduce LDH levels; enhance HO-1 and NQO1 expression	↓: ROS, LDH; ↑: *hmox1* (HO-1), *nqo1*, Nrf2 pathway activation	(79)
	*Forsythia suspensa*	Forsythiaside A (FA)	Acetaminophen (APAP)-induced liver injury [*Tg(lfabp: EGFP)* zebrafish]	Modulate extracellular matrix remodeling; inhibit PI3K/AKT-mediated apoptosis; restore GSH content	↓: ALT, AST, apoptosis; ↑: GSH; PI3K/AKT pathway modulation	(119)
	*Glycyrrhiza*	Isoliquiritigenin (ISL)	EMO-induced liver injury in the zebrafish larvae *Tg (L-FABP: EGFP)*	Activate Nrf2 signaling; reduce ROS and MDA; enhance GSH and SOD	↓: ROS, MDA; ↑: GSH, SOD, Nrf2 pathway activation	(120)
	*Hedyotis diffusa* Willd.	ethanol extract (HDWE)	Isoniazid (INH)-induced liver injury [*Tg(L-FABP: EGFP)* larvae]	Reverse INH-induced hepatotoxicity;	↓: INH-induced cytotoxicity; ↑: Cell viability	(121)
Liver protection	Shen Quan baijiu (SQJ)	ginsenoside Rg5/Rk1	Alcoholic liver disease (ALD) zebrafish model	Antioxidant; enhance SOD and GSH-Px activities; reduce MDA production	↑: GSH-Px, SOD; ↓: MDA	(72)

These studies use wild-type and transgenic zebrafish as experimental models to provide information on the protective effects of natural product extracts or monomeric components against cardiac and hepatic injury induced by chemical toxicants (e.g., verapamil, aristolochic acid, thioacetamide, acetaminophen, emodin, isoniazid, ethanol). “↑” indicates increase, upregulation, or promotion; “↓” indicates decrease, downregulation, or inhibition; “↔” indicates no significant change.

### 4.1 Cardiac repair and protection: harnessing zebrafish regeneration to combat toxicity

The zebrafish heart possesses a remarkable innate capacity for regeneration, a trait that provides a unique window into mechanisms of cardiac repair following injury. Landmark studies established that adult zebrafish can fully regenerate up to 20% of the ventricular myocardium, a process driven by the dedifferentiation and proliferation of existing cardiomyocytes orchestrated by conserved signaling pathways including FGF, TGF-*β*, and BMP (63, 64). This regenerative paradigm is now leveraged in models of chemical cardiotoxicity, such as those induced by aristolochic acid or pharmacologic agents like verapamil, which mimic aspects of heart failure, arrhythmia, and hypertrophy (65, 66). These models are instrumental for probing how interventions can modulate key injury responses like inflammation, fibrosis, and cell death (Table 3).

Research utilizing these models has identified several plant-derived compounds with cardioprotective potential. A bioactive component from water chestnuts attenuated inflammation and pathological hypertrophy in an aristolochic acid-induced model by suppressing MAPK signaling (67). Similarly, a suite of ginsenosides and organic acids from American ginseng alleviated verapamil/digoxin-induced heart failure phenotypes, reducing pericardial edema and improving cardiac function (68). The zebrafish model also elegantly reveals the dose-dependent duality of some natural agents. Artesunate from *Artemisia annua*, for example, proved cardioprotective at low doses but induced toxicity at higher concentrations, highlighting the importance of therapeutic window assessment (69). Furthermore, compounds like the flaxseed lignan secoisolariciresinol have shown promise in preventing toxicant-induced pericardial edema, suggesting a role in maintaining normal cardiac morphology under stress (70).

### 4.2 Hepatic defense and regeneration: counteracting chemical insults in the zebrafish liver

The zebrafish liver shares substantial anatomical, functional, and regenerative homology with its human counterpart, making it a powerful model for hepatotoxicity studies and the discovery of hepatoprotective agents (71). A variety of chemical agents—including thioacetamide (TAA), acetaminophen (APAP), ethanol, and isoniazid (INH)—are used to induce liver injury, modeling conditions from alcoholic liver disease (ALD) and drug-induced liver injury to steatosis and fibrosis. These models enable the evaluation of herbal compounds across a spectrum of pathological hallmarks, including oxidative stress, inflammation, lipid accumulation, and apoptosis (Table 3).

Numerous plant extracts and purified compounds have demonstrated potent liver-protective effects in these zebrafish systems. Against alcoholic liver disease, ginsenosides Rg5/Rk1 enhanced the activity of endogenous antioxidant enzymes (SOD, GSH-Px) and reduced lipid peroxidation, thereby improving ethanol metabolism and alleviating oxidative damage (72). In TAA-induced injury models, a range of compounds exerted protection through multifaceted mechanisms. Extracts of *Salvia plebeia* and processed *Polygonum cuspidatum* reduced inflammation, steatosis, and fibrosis, with the latter acting through inhibition of the TLR4/MyD88/NF-κB pathway (73, 74). The phenylpropanoid chlorogenic acid promoted liver growth and reduced apoptosis, effects that were accompanied by the modulation of Wnt/*β*-catenin pathway-related gene expression (75), while flavonoids like baicalein, rhamnetin 3-O-*α*-rhamnoside (ARR), and hydroxysafflor yellow A (HSYA) attenuated damage by targeting oxidative stress and inflammatory signaling through MAPK and IKK*β*/NF-κB pathways (47, 76–78). Similarly, in APAP-induced toxicity models, compounds such as chebulinic acid and forsythiaside A exhibited protection by activating the Nrf2 antioxidant pathway and inhibiting apoptosis (79, 119). These collective findings underscore the zebrafish liver’s utility as a holistic *in vivo* platform for validating the hepatoprotective efficacy of herbal medicines and deciphering their complex, often multi-targeted, mechanisms of action against chemical insults. Collectively, these hepatic studies exemplify the protective paradigm: plant-derived compounds preserve hepatocyte viability and organ function by activating antioxidant defenses and inhibiting apoptosis, rather than by stimulating cell proliferation.

## 5 Synthesis and future directions: integrating zebrafish models into the discovery of plant-based regenerative therapies

**Figure 2:**
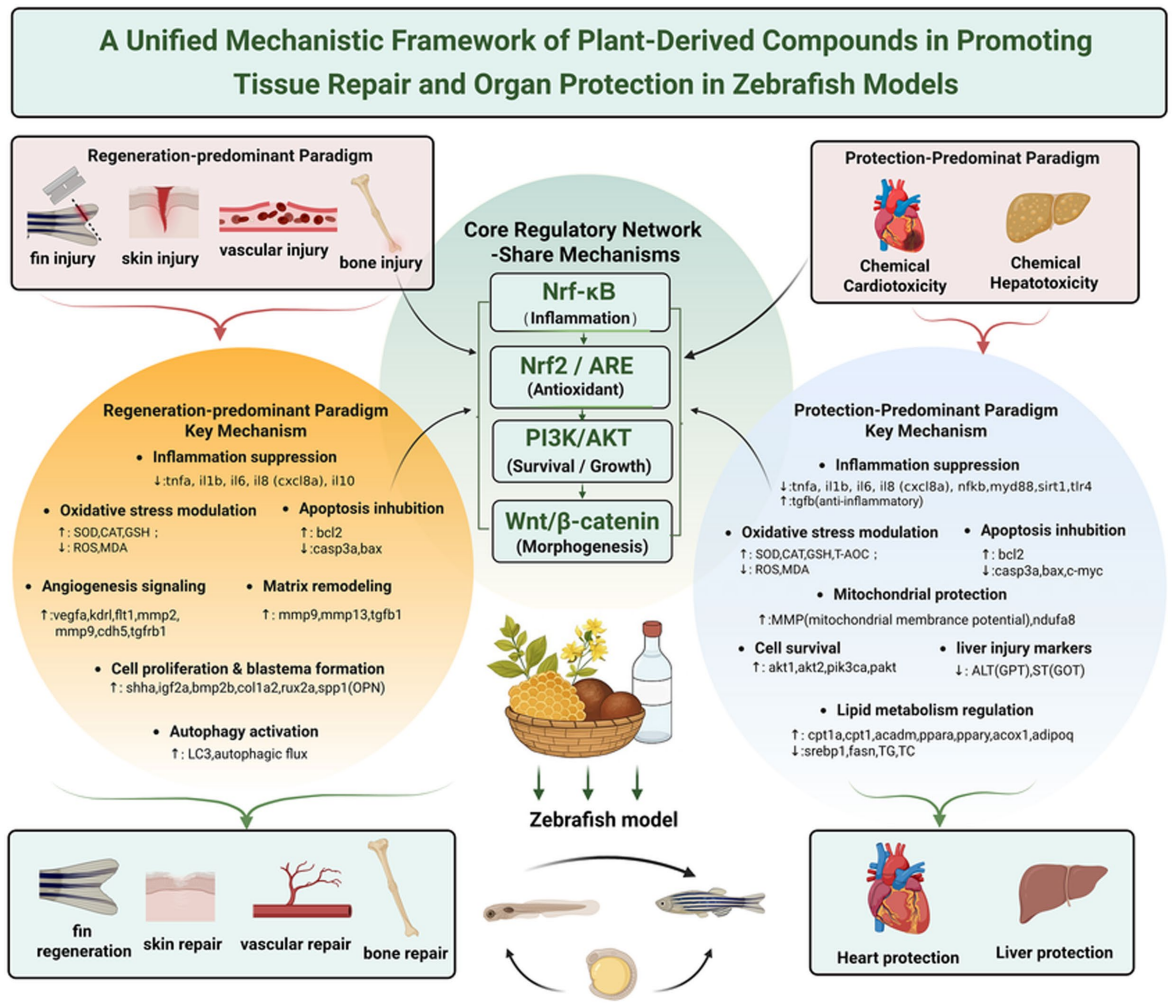
A unified mechanistic framework of plant-derived compounds in promoting tissue repair and organ protection in zebrafish models. The schematic integrates the two major paradigms identified in this review. The left panel (Regeneration-Predominant Paradigm) summarizes the key mechanisms underlying *de novo* tissue formation in fin, skin, bone, and vascular injury models, including inflammation suppression, oxidative stress modulation, apoptosis inhibition, angiogenesis signaling, cell proliferation and blastema formation, and autophagy activation. The right panel (Protection-Predominant Paradigm) summarizes the mechanisms mitigating chemical-induced cardiac and hepatic damage, including inflammation suppression, oxidative stress modulation, apoptosis inhibition, mitochondrial protection, and lipid metabolism regulation. The central area highlights the core regulatory network—NF-κB (inflammation), Nrf2/ARE (antioxidant), PI3K/AKT (survival/growth), and Wnt/*β*-catenin (morphogenesis)—that constitutes the evolutionarily conserved signaling architecture repeatedly targeted by plant-derived compounds across all injury contexts. Representative gene and indicator changes (↑, upregulation/increase; ↓, downregulation/decrease) are listed for each mechanism within each paradigm. This figure was created in BioRender. Meng, A. (2026) https://BioRender.com/qdp6bwl and is published under agreement No. QI29OZ121O.

This review has synthesized compelling evidence that the zebrafish (*Danio rerio*) serves as an indispensable *in vivo* platform for evaluating and deconvoluting the therapeutic potential of plant-derived compounds in tissue repair. As summarized in Figure 2, a diverse array of injury models—encompassing external wounds (caudal fin, skin), internal tissue damage (bone, vasculature), and chemical-induced organ injury (heart, liver)—provide a versatile toolkit. These models, combined with transgenic lines and molecular readouts, allow researchers to move beyond simple phenotypic observation to mechanistic dissection.

A striking observation emerging from this body of evidence is that plant-derived compounds with diverse chemical structures and botanical origins converge on a remarkably limited set of signaling hubs to exert their pro-repair effects across vastly different injury contexts. Regardless of whether the endpoint is fin regeneration, bone mineralization, vascular repair, or hepatoprotection, the same functional nodes—NF-κB, Nrf2, PI3K/AKT, and Wnt/*β*-catenin—are repeatedly implicated (Tables 1–3). This convergence is unlikely to be coincidental. Rather, it reflects the existence of an evolutionarily conserved “core regulatory network” that governs the cellular decision between stress-induced apoptosis and survival-associated regeneration. In regeneration-predominant contexts (fin, bone, vasculature), plant compounds activate PI3K/AKT and Wnt/β-catenin to drive proliferation and morphogenesis while fine-tuning NF-κB to permit necessary inflammation without allowing its escalation. In protection-predominant contexts (liver, heart), the same compounds prioritize Nrf2-driven antioxidant defenses and direct inhibition of NF-κB and apoptosis, preserving tissue integrity until endogenous repair mechanisms can engage. This unified framework explains why so many chemically unrelated phytochemicals exhibit remarkably similar pro-repair spectra: they have been shaped by evolution to target the same ancient stress-response architecture that is conserved from zebrafish to humans.

The aggregated data, summarized in Tables 1–3, demonstrate that herbal extracts and their active constituents exert pro-regenerative effects by modulating a network of evolutionarily conserved pathways. Crucially, these pathways—mediating inflammation, oxidative stress, autophagy, and apoptosis—do not operate in isolation but engage in extensive and context-dependent crosstalk that underpins both *de novo* tissue regeneration and the mitigation of chemical-induced organ damage. In the canonical repair sequence, an initial burst of reactive oxygen species (ROS) following injury acts as a potent signaling trigger, simultaneously initiating the inflammatory cascade and the autophagic machinery (85). Early, moderate inflammation is essential for clearing cellular debris and activating regenerative programs, while autophagy plays a dual role: it removes damaged mitochondria to curb excessive ROS generation and provides metabolic substrates for proliferating cells. However, when ROS overwhelm endogenous antioxidant defenses, they hyperactivate the NF-κB pathway, amplifying the release of pro-inflammatory mediators and driving a vicious cycle of escalating oxidative damage and chronic inflammation. This unresolved cycle, together with sustained autophagic impairment, converges on the activation of the intrinsic apoptotic pathway, culminating in irreversible cell loss and fibrotic scarring. Phytochemicals inherently target multiple nodes within this integrated network simultaneously. For instance, compounds that activate the Nrf2 pathway, such as isoquercitrin and hydroxysafflor yellow A (Table 2), upregulate antioxidant enzymes to suppress ROS and, consequently, attenuate the downstream inflammatory cascade. Concurrently, bioactive molecules and botanical drugs like betulin and *Radix Rehmanniae Praeparata* (Table 1) can both dampen NF-κB-mediated inflammation and enhance autophagic flux, thereby interrupting the feed-forward loop that precipitates apoptosis. This capacity to synergistically recalibrate the ROS–inflammation–autophagy–apoptosis axis, rather than merely antagonizing a single target, is a defining feature of phytopharmacology and the molecular basis underlying their promotion of tissue repair.

These mechanistic insights, while compelling, should be interpreted in the context of the methodological limitations that characterize much of the current evidence base. While the collective evidence summarized in Tables 1–3 provides compelling support for the pro-repair effects of plant-derived compounds, several methodological limitations common to this body of literature warrant explicit acknowledgment. Most of the reviewed studies employed relatively small sample sizes without reporting statistical power calculations or effect sizes, and the choice of positive controls varied considerably—some using established agents such as beclomethasone or 17β-estradiol, others relying solely on untreated vehicle controls—making cross-study comparisons of relative efficacy difficult. Dose selection was frequently empirical, based on prior publications or pilot assays, and few studies characterized pharmacokinetic profiles or defined therapeutic windows, a concern underscored by the dose-dependent dual effects documented for compounds such as artesunate and Rg1, where protective actions at low concentrations give way to toxicity at higher doses. Furthermore, mechanistic conclusions were often drawn primarily from mRNA expression changes, with comparatively fewer investigations providing corroborating protein-level evidence or functional pathway validation using specific inhibitors or genetic mutants. It should be emphasized that gene expression analysis alone, while valuable for generating mechanistic hypotheses, constitutes only preliminary evidence of pathway involvement; establishing a functional role for a given signaling cascade requires orthogonal approaches such as pharmacological blockade (e.g., using G15, LY294002, or other pathway-specific inhibitors), genetic perturbation (e.g., morpholino knockdown, CRISPR/Cas9 mutagenesis, or the use of existing mutant lines such as gr^s357), or rescue experiments. Future studies would benefit from adopting such multi-level validation strategies to move beyond correlative observations and toward definitive mechanistic conclusions. Similarly, the majority of studies did not report whether randomization, blinding, or pre-registered protocols were employed, practices that are essential for minimizing observer and selection bias. A final conceptual issue is that several studies grouped under “tissue repair” actually evaluate protection against acute chemical-induced organ damage rather than *de novo* regeneration, a distinction that affects how endpoints should be interpreted. These methodological gaps do not diminish the value of the findings reviewed herein but highlight clear opportunities for refinement.

Several physiological and practical factors limit direct extrapolation of zebrafish findings to humans. In most larval studies, phytochemicals are administered by immersion, bypassing the oral absorption and first-pass hepatic metabolism that occur in humans, and the functional conservation of zebrafish CYP enzymes involved in phytochemical biotransformation remains poorly characterized. The immune system of larval zebrafish also differs substantially, lacking the adaptive immune components that shape repair outcomes in adult mammals. Moreover, effective concentrations are rarely converted to mammalian equivalent doses using allometric scaling, making it difficult to assess whether therapeutic effects observed in zebrafish fall within a clinically achievable range—a concern highlighted by the dose-dependent dual effects of compounds such as artesunate and Rg1.

Despite these challenges, the zebrafish model continues to evolve toward greater translation relevance. Furthermore, while excellent for studying acute injury and regeneration, modeling chronic human conditions—such as non-healing diabetic ulcers, age-related degenerative diseases, or multifactorial metabolic syndromes—remains a developing area (90). Notably, significant progress has been made in establishing zebrafish models that recapitulate key aspects of chronic human pathologies. For instance, prolonged high-fat diet feeding in adult zebrafish reliably induces obesity, hyperglycemia, and hepatic steatosis that can progress to non-alcoholic steatohepatitis (NASH) with fibrosis, mimicking the trajectory of human metabolic syndrome. Similarly, aging zebrafish colonies have been established to study age-dependent decline in fin and cardiac regeneration, providing a platform to test whether phytochemicals can restore regenerative capacity in aged organisms. These chronic models, however, have not yet been widely adopted for screening plant-derived compounds, as the vast majority of studies reviewed herein employed acute injury paradigms in larvae or young adults. Extending phytochemical screening to these chronic and aging models represents a promising direction for future research, as it would enable the evaluation of natural products under conditions that more faithfully reflect the complex, long-term pathophysiology of human degenerative and metabolic diseases. Current models often focus on single tissues; developing more sophisticated paradigms that capture multi-organ interactions or the impact of systemic comorbidities on healing would enhance translational relevance (122).

Looking forward, future research should also prioritize the investigation of individual variables like age, sex, and genetic background, which significantly influence repair outcomes and therapeutic efficacy, to pave the way for personalized regenerative strategies. Age is a particularly critical factor, as the regenerative capacity of zebrafish declines substantially from larval to adult stages. Larval zebrafish heal rapidly and scarlessly, whereas adults heal more slowly and exhibit a greater propensity for fibrotic scarring (37). Similarly, sex-dependent differences have been documented: as discussed in Section 2.1, male zebrafish regenerate their caudal fins more slowly than females under basal conditions, and the dose–response relationship to phytochemical treatment can differ markedly between sexes (35, 123). These age- and sex-related variables are likely mediated through differential regulation of inflammatory responses, hormonal signaling, and cellular proliferation rates. In addition to these physiological factors, genetic background plays a pivotal role. For instance, the differential regenerative capacity observed between wild-type strains (e.g., AB versus TU) underscores the role of inherent genetic variation in determining repair competence. Studies employing glucocorticoid receptor (gr) mutant zebrafish have directly demonstrated that a single genetic alteration can abolish the anti-inflammatory and regenerative effects of ginsenoside Rg1, highlighting how individual genetic differences in key signaling pathways can dictate therapeutic responsiveness. Equallly important, the dose–response relationship itself warrants focused investigation, as several compounds reviewed herein exhibit dose-dependent dual effects. Artesunate provides an instructive example: at low doses it significantly restored cardiac function in a verapamil-induced heart failure model, whereas at higher doses the same compound induced cardiotoxicity, nephrotoxicity, and developmental toxicity in larval zebrafish (69). Similar biphasic behavior has been documented for ginsenoside Rg1, which promotes bone formation at moderate concentrations but causes developmental malformations at elevated doses (46), and suppresses inflammation without impairing fin regeneration only within a defined concentration range (29). This pattern—beneficial at low doses, harmful at high doses—is not merely a toxicological caveat but a defining feature of many phytochemicals. Systematically characterizing the dose–response landscape for each compound and establishing allometrically scaled mammalian equivalent doses will be essential for translating zebrafish-based discoveries toward clinical applications.

The combined use of zebrafish and herbal medicine has proven highly effective for initial screening and mechanistic hypothesis generation. The model’s strengths—including optical transparency for real-time imaging, high fecundity for throughput, and strong genetic tractability—are particularly well-suited to studying the polypharmacology inherent to plant extracts (80). Emerging technologies promise to deepen these insights further. CRISPR/Cas9 gene editing enables the creation of precise disease models and the functional validation of drug targets within repair pathways (16). Advanced live imaging techniques, including light-sheet and two-photon microscopy, will allow unprecedented visualization of cellular behaviors and cell–cell interactions during compound-mediated healing (81). When integrated with omics technologies, these approaches can rapidly capture the systems-level biological activities of complex botanicals, accelerating the identification of novel bioactive principles (86).

Notwithstanding these considerations, the evidence consolidated in this review unequivocally establishes the zebrafish as a pre-eminent and versatile platform in natural product research for tissue repair. It successfully bridges high-throughput discovery with deep mechanistic inquiry, translating the complex pharmacology of plant extracts into understandable and actionable insights into regeneration. Addressing the aforementioned limitations represents not a weakness of the model, but the clear and exciting pathway for its evolving application in translational regenerative medicine.

## 6 Conclusion

In conclusion, the zebrafish model provides a powerful and ethically favorable bridge between traditional phytomedicine and modern mechanistic biology. It provides *in vivo* validation of the health effects of natural compounds—specifically their capacity to promote tissue repair and regeneration. The evidence reviewed herein underscores that these health effects are not anecdotal but are rooted in the modulation of evolutionarily conserved repair mechanisms. Looking forward, the continued refinement of zebrafish injury models, coupled with cutting-edge genetic and imaging tools, will significantly enhance our ability to discover, optimize, and understand plant-based therapeutics. By faithfully mirroring key aspects of human disease and healing while offering unmatched observational power, the zebrafish stands as a cornerstone model in the accelerating quest to translate the regenerative potential of the plant kingdom into tangible clinical benefits for human health.

## References

[1] Eming SA Martin P Tomic-Canic M. Wound repair and regeneration: mechanisms, signaling, and translation. Sci Transl Med. (2014) 6:265sr6. doi: 10.1126/scitranslmed.3009337, 25473038 PMC4973620

[2] Ji S Xiong M Chen H Liu Y Zhou L Hong Y . Cellular rejuvenation: molecular mechanisms and potential therapeutic interventions for diseases. Signal Transduct Target Ther. (2023) 8:116. doi: 10.1038/s41392-023-01343-5, 36918530 PMC10015098

[3] Guo S Dipietro LA. Factors affecting wound healing. J Dent Res. (2010) 89:219–29. doi: 10.1177/0022034509359125, 20139336 PMC2903966

[4] Loi F Córdova LA Pajarinen J Lin T-H Yao Z Goodman SB. Inflammation, fracture and bone repair. Bone. (2016) 86:119–30. doi: 10.1016/j.bone.2016.02.020, 26946132 PMC4833637

[5] Kang W Robitaille MC Merrill M Teferra K Kim C Raphael MP. Mechanisms of cell damage due to mechanical impact: an *in vitro* investigation. Sci Rep. (2020) 10:12009. doi: 10.1038/s41598-020-68655-2, 32686715 PMC7371734

[6] Leslie J Geh D Elsharkawy AM Mann DA Vacca M. Metabolic dysfunction and cancer in HCV: shared pathways and mutual interactions. J Hepatol. (2022) 77:219–36. doi: 10.1016/j.jhep.2022.01.029, 35157957

[7] Vilas-Boas EA Almeida DC Roma LP Ortis F Carpinelli AR. Lipotoxicity and β-cell failure in type 2 diabetes: oxidative stress linked to NADPH oxidase and ER stress. Cells. (2021) 10:3328. doi: 10.3390/cells10123328, 34943836 PMC8699655

[8] Lv X Zhao T Dai Y Shi M Huang X Wei Y . New insights into the interplay between autophagy and cartilage degeneration in osteoarthritis. Front Cell Dev Biol. (2022) 10:1089668. doi: 10.3389/fcell.2022.1089668, 36544901 PMC9760856

[9] Fan J-S Liu D-N Huang G Xu Z-Z Jia Y Zhang H-G . Panax notoginseng saponins attenuate atherosclerosis via reciprocal regulation of lipid metabolism and inflammation by inducing liver X receptor alpha expression. J Ethnopharmacol. (2012) 142:732–8. doi: 10.1016/j.jep.2012.05.053, 22683903

[10] Jebur AB El-Sayed RA Abdel-Daim MM El-Demerdash FM. *Punica granatum* (pomegranate) Peel extract pre-treatment alleviates Fenpropathrin-induced testicular injury via suppression of oxidative stress and inflammation in adult male rats. Toxics. (2023) 11:504. doi: 10.3390/toxics11060504, 37368604 PMC10301163

[11] Cheng TO. Cardiovascular effects of Danshen. Int J Cardiol. (2007) 121:9–22. doi: 10.1016/j.ijcard.2007.01.004, 17363091

[12] Grabarczyk M Justyńska W Czpakowska J Smolińska E Bielenin A Glabinski A . Role of plant phytochemicals: resveratrol, curcumin, luteolin and quercetin in demyelination, neurodegeneration, and epilepsy. Antioxidants. (2024) 13:1364. doi: 10.3390/antiox13111364, 39594506 PMC11591432

[13] Fu Z Zhao P-Y Yang X-P Li H Hu S-D Xu Y-X . Cannabidiol regulates apoptosis and autophagy in inflammation and cancer: a review. Front Pharmacol. (2023) 14:1094020. doi: 10.3389/fphar.2023.1094020, 36755953 PMC9899821

[14] Ravikumar B Aittokallio T. Improving the efficacy-safety balance of polypharmacology in multi-target drug discovery. Expert Opin Drug Discov. (2017) 13:179–92. doi: 10.1080/17460441.2018.1413089, 29233023

[15] Beffagna G. Zebrafish as a smart model to understand regeneration after heart injury: how fish could help humans. Front Cardiovasc Med. (2019) 6:107. doi: 10.3389/fcvm.2019.00107, 31448289 PMC6691037

[16] Marques IJ Lupi E Mercader N. Model systems for regeneration: zebrafish. Development. (2019) 146:167692. doi: 10.1242/dev.167692, 31540899

[17] Bauer B Mally A Liedtke D. Zebrafish embryos and larvae as alternative animal models for toxicity testing. Int J Mol Sci. (2021) 22:13417. doi: 10.3390/ijms222413417, 34948215 PMC8707050

[18] Li Y Jia Z Zhang S He X. Progress in gene-editing Technology of Zebrafish. Biomolecules. (2021) 11:1300. doi: 10.3390/biom11091300, 34572513 PMC8468279

[19] Lebert DC Huttenlocher A. Inflammation and wound repair. Semin Immunol. (2014) 26:315–20. doi: 10.1016/j.smim.2014.04.007, 24853879 PMC6801000

[20] Li J Wen W Zhang S Zhou C Feng Y Li X. The expression and function of lincRNA-154324 and the adjoining protein-coding gene vmp1 in the caudal fin regeneration of zebrafish. Int J Mol Sci. (2022) 23:8944. doi: 10.3390/ijms23168944, 36012210 PMC9409064

[21] Rabinowitz JS Robitaille AM Wang Y Ray CA Thummel R Gu H . Transcriptomic, proteomic, and metabolomic landscape of positional memory in the caudal fin of zebrafish. Proc Natl Acad Sci. (2017) 114:E717–26. doi: 10.1073/pnas.1620755114, 28096348 PMC5293114

[22] Hagedorn M Siegfried G Hooks KB Khatib AM. Integration of zebrafish fin regeneration genes with expression data of human tumors *in silico* uncovers potential novel melanoma markers. Oncotarget. (2016) 7:71567–79. doi: 10.18632/oncotarget.12257, 27689402 PMC5342102

[23] Miskolci V Squirrell J Rindy J Vincent W Sauer JD Gibson A . Distinct inflammatory and wound healing responses to complex caudal fin injuries of larval zebrafish. eLife. (2019) 8:45976. doi: 10.7554/eLife.45976, 31259685 PMC6602581

[24] Pfefferli C Jaźwińska A. The art of fin regeneration in zebrafish. Regeneration. (2015) 2:72–83. doi: 10.1002/reg2.33, 27499869 PMC4895310

[25] Uemoto T Abe G Tamura K. Regrowth of zebrafish caudal fin regeneration is determined by the amputated length. Sci Rep. (2020) 10:649. doi: 10.1038/s41598-020-57533-6, 31959817 PMC6971026

[26] Duarte Da Silva KC Carneiro WF Virote BDCR Santos MDF De Oliveira JPL Castro TFD . Evaluation of the anti-inflammatory and antioxidant potential of *Cymbopogon citratus* essential oil in zebrafish. Animals. (2024) 14:581. doi: 10.3390/ani14040581, 38396549 PMC10886050

[27] Xu S Zhang H Li C-Z Lai P-S Wang G Chan YS . Cannabidiol promotes fin regeneration and reduces apoptosis in zebrafish embryos. J Funct Foods. (2021) 86:104694. doi: 10.1016/j.jff.2021.104694

[28] Martínez-Llorens S Liu W Yu H Gurbazar D Rinchindorj D Kang W . Anti-inflammatory effects and beneficial effects of the feed additive *Urtica cannabina* L. in zebrafish. PLoS One. (2024) 19:e0307269. doi: 10.1371/journal.pone.0307269 39018284 PMC11253947

[29] He M Halima M Xie Y Schaaf MJM Meijer AH Wang M. Ginsenoside Rg1 acts as a selective glucocorticoid receptor agonist with anti-inflammatory action without affecting tissue regeneration in zebrafish larvae. Cells. (2020) 9:1107. doi: 10.3390/cells9051107, 32365641 PMC7290513

[30] Long Y Zhao T Xiao Y Kong S Wang R Cai K . Effect of oxymatrine on neutrophil function based on zebrafish inflammation model and primary neutrophil inflammatory responses. Int Immunopharmacol. (2024) 142:113064. doi: 10.1016/j.intimp.2024.113064, 39243560

[31] Ouyang T Yin H Yang J Liu Y Ma S. Tissue regeneration effect of betulin via inhibition of ROS/MAPKs/NF-ĸB axis using zebrafish model. Biomed Pharmacother. (2022) 153:113420. doi: 10.1016/j.biopha.2022.113420, 36076542

[32] Chen F Pu S Tian L Zhang H Zhou H Yan Y . Radix Rehmanniae Praeparata promoted zebrafish fin regeneration through aryl hydrocarbon receptor-dependent autophagy. J Ethnopharmacol. (2024) 331:118272. doi: 10.1016/j.jep.2024.118272, 38710459

[33] Cheng J Fu Y Meng X Tang G Li L Yusupov Z . Investigation of anti-inflammatory effect of essential oil extracted from *Achillea alpina* L. through multi-omics analysis in zebrafish tail fin amputation model. J Ethnopharmacol. (2025) 344:119519. doi: 10.1016/j.jep.2025.119519, 39986357

[34] Wibowo I Utami N Anggraeni T Barlian A Putra RE Indriani AD . Propolis can improve caudal fin regeneration in zebrafish (*Danio rerio*) induced by the combined administration of alloxan and glucose. Zebrafish. (2021) 18:274–81. doi: 10.1089/zeb.2020.1969 34297614

[35] Zainol Abidin IZ Fazry S Jamar NH Ediwar Dyari HR Zainal Ariffin Z Johari AN . The effects of *Piper sarmentosum* aqueous extracts on zebrafish (*Danio rerio*) embryos and caudal fin tissue regeneration. Sci Rep. (2020) 10:14165. doi: 10.1038/s41598-020-70962-7, 32843675 PMC7447815

[36] Richardson R Slanchev K Kraus C Knyphausen P Eming S Hammerschmidt M. Adult zebrafish as a model system for cutaneous wound-healing research. J Invest Dermatol. (2013) 133:1655–65. doi: 10.1038/jid.2013.16, 23325040 PMC3644348

[37] Naomi R Bahari H Yazid MD Embong H Othman F. Zebrafish as a model system to study the mechanism of cutaneous wound healing and drug discovery: advantages and challenges. Pharmaceuticals. (2021) 14:1058. doi: 10.3390/ph14101058, 34681282 PMC8539578

[38] Zain MSC Edirisinghe SL Kim C-H De Zoysa M Shaari K. Nanoemulsion of flavonoid-enriched oil palm (*Elaeis guineensis* Jacq.) leaf extract enhances wound healing in zebrafish. Phytomed Plus. (2021) 1:100124. doi: 10.1016/j.phyplu.2021.100124

[39] Athiroh N Hayati A Pudjiwati I Taufiq A Mubarakati NJ. The portrait of neem leaves-based high performance wound healing activity on zebrafish. Berkala Penelit Hayati. (2021) 27:23–7. doi: 10.23869/bphjbr.27.1.20214

[40] Dalle Carbonare L Braggio M Minoia A Cominacini M Romanelli MG Pessoa J . Modeling musculoskeletal disorders in zebrafish: advancements in muscle and bone research. Cells. (2024) 14:28. doi: 10.3390/cells14010028, 39791729 PMC11719663

[41] Mackay EW Apschner A Schulte-Merker S. A bone to pick with zebrafish. BoneKEy Reports. (2013) 2:445. doi: 10.1038/bonekey.2013.179, 24422140 PMC3844975

[42] Wu Z Li W Jiang K Lin Z Qian C Wu M . Regulation of bone homeostasis: signaling pathways and therapeutic targets. MedComm. (2024) 5:e657. doi: 10.1002/mco2.657, 39049966 PMC11266958

[43] Dietrich K Fiedler IaK Kurzyukova A López-Delgado AC Mcgowan LM Geurtzen K . Skeletal biology and disease modeling in zebrafish. J Bone Miner Res. (2020) 36:436–58. doi: 10.1002/jbmr.4256 33484578

[44] Geurtzen K Knopf F Wehner D Huitema LFA Schulte-Merker S Weidinger G. Mature osteoblasts dedifferentiate in response to traumatic bone injury in the zebrafish fin and skull. Development. (2014) 141:2225–34. doi: 10.1242/dev.105817, 24821985

[45] Carnovali M Ramoni G Banfi G Mariotti M. Herbal preparation (bromelain, papain, Curcuma, black pepper) enhances mineralization and reduces glucocorticoid-induced osteoporosis in zebrafish. Antioxidants. (2021) 10:1987. doi: 10.3390/antiox10121987, 34943090 PMC8750159

[46] Jiang Z Deng L Li M Alonge E Wang Y Wang Y. Ginsenoside Rg1 modulates PI3K/AKT pathway for enhanced osteogenesis via GPER. Phytomedicine. (2024) 124:155284. doi: 10.1016/j.phymed.2023.155284, 38176267

[47] Zhao Y Wang R Li A Zhao P Yang J. Protective effect of hydroxysafflor yellow a on thioacetamide-induced liver injury and osteopenia in zebrafish. Toxicol Appl Pharmacol. (2024) 492:117109. doi: 10.1016/j.taap.2024.117109, 39306099

[48] Li X Zhou D Yang D Fu Y Tao X Hu X . Isoquercitrin attenuates osteogenic injury in MC3T3 osteoblastic cells and the zebrafish model via the Keap1-Nrf2-ARE pathway. Molecules. (2022) 27:3459. doi: 10.3390/molecules27113459, 35684398 PMC9182080

[49] Johnson KE Wilgus TA. Vascular endothelial growth factor and angiogenesis in the regulation of cutaneous wound repair. Adv Wound Care. (2014) 3:647–61. doi: 10.1089/wound.2013.0517, 25302139 PMC4183920

[50] Gore AV Monzo K Cha YR Pan W Weinstein BM. Vascular development in the zebrafish. Cold Spring Harb Perspect Med. (2012) 2:a006684–4. doi: 10.1101/cshperspect.a006684, 22553495 PMC3331685

[51] Hasnat H Shompa SA Islam MM Alam S Richi FT Emon NU . Flavonoids: a treasure house of prospective pharmacological potentials. Heliyon. (2024) 10:e27533. doi: 10.1016/j.heliyon.2024.e27533, 38496846 PMC10944245

[52] Marino A Battaglini M Moles N Ciofani G. Natural antioxidant compounds as potential pharmaceutical tools against neurodegenerative diseases. ACS Omega. (2022) 7:25974–90. doi: 10.1021/acsomega.2c03291, 35936442 PMC9352343

[53] Yuan M Zhang G Bai W Han X Li C Bian S . The role of bioactive compounds in natural products extracted from plants in Cancer treatment and their mechanisms related to anticancer effects. Oxidative Med Cell Longev. (2022) 2022:1–19. doi: 10.1155/2022/1429869, 35211240 PMC8863487

[54] Ho T-J Tsai P-H Hsieh C-H Lin J-H Lin Y-W Wu J-R . Role of herbal extracts of catechu from *Uncaria gambir* in the treatment of chronic diabetic wounds. Pharmaceuticals. (2022) 16:66. doi: 10.3390/ph16010066, 36678562 PMC9863412

[55] Wang Y Wang S Wang Y Gao P Wang L Wang Q . The natural compound sinometumine E derived from Corydalis decumbens promotes angiogenesis by regulating HIF-1/ VEGF pathway in vivo and in vitro. Biomed Pharmacother. (2024) 178:117113. doi: 10.1016/j.biopha.2024.117113, 39067164

[56] Hu G Mahady GB Li S Hoi MP Wang YH Lee SM. Polysaccharides from astragali radix restore chemical-induced blood vessel loss in zebrafish. Vasc Cell. (2012) 4:2. doi: 10.1186/2045-824x-4-2, 22357377 PMC3316134

[57] Zhou Q Ma J Liu Q Wu C Yang Z Yang T . Traditional Chinese medicine formula, Sanwujiao granule, attenuates ischemic stroke by promoting angiogenesis through early administration. J Ethnopharmacol. (2024) 321:117418. doi: 10.1016/j.jep.2023.117418, 37979814

[58] Giordo R Nasrallah GK Al-Jamal O Paliogiannis P Pintus G. Resveratrol inhibits oxidative stress and prevents mitochondrial damage induced by zinc oxide nanoparticles in zebrafish (*Danio rerio*). Int J Mol Sci. (2020) 21:3838. doi: 10.3390/ijms21113838, 32481628 PMC7312482

[59] Lam HW Lin HC Lao SC Gao JL Hong SJ Leong CW . The angiogenic effects of Angelica sinensis extract on HUVEC in vitro and zebrafish *in vivo*. J Cell Biochem. (2007) 103:195–211. doi: 10.1002/jcb.21403, 17497682

[60] Liu C-L Cheng L Kwok H-F Ko C-H Lau T-W Koon C-M . Bioassay-guided isolation of norviburtinal from the root of Rehmannia glutinosa, exhibited angiogenesis effect in zebrafish embryo model. J Ethnopharmacol. (2011) 137:1323–7. doi: 10.1016/j.jep.2011.07.060, 21843616

[61] Wang J Zhang X-H Xu X Zhu Q Yao B Liang S . Pro-angiogenic activity of Tongnao decoction on HUVECs *in vitro* and zebrafish *in vivo*. J Ethnopharmacol. (2020) 254:112737. doi: 10.1016/j.jep.2020.112737, 32147480

[62] Yu X Tong Y Kwok HF Sze SC Zhong L Lau CB . Anti-angiogenic activity of erxian decoction, a traditional Chinese herbal formula, in zebrafish. Biol Pharm Bull. (2012) 35:2119–27. doi: 10.1248/bpb.b12-00130, 23018578

[63] Gonzalez-Rosa JM Burns CE Burns CG. Zebrafish heart regeneration: 15 years of discoveries. Regeneration. (2017) 4:105–23. doi: 10.1002/reg2.83, 28979788 PMC5617908

[64] Poss KD Wilson LG Keating MT. Heart regeneration in zebrafish. Science. (2002) 298:2188–90. doi: 10.1126/science.1077857 12481136

[65] Buja LM. Pathobiology of myocardial and cardiomyocyte injury in ischemic heart disease: perspective from seventy years of cell injury research. Exp Mol Pathol. (2024) 140:104944. doi: 10.1016/j.yexmp.2024.104944, 39577392

[66] Guo Q Wang J Ni C Pan J Zou J Shi Y . Research progress on the natural products in the intervention of myocardial infarction. Front Pharmacol. (2024) 15:1445349. doi: 10.3389/fphar.2024.1445349, 39239656 PMC11374734

[67] Haege ER Huang HC Huang CC. Identification of lactate as a cardiac protectant by inhibiting inflammation and cardiac hypertrophy using a zebrafish acute heart failure model. Pharmaceuticals (Basel). (2021) 14:261. doi: 10.3390/ph14030261, 33803943 PMC7999541

[68] Dong R Zhang Y Chen S Wang H Hu K Zhao H . Identification of key pharmacodynamic markers of American ginseng against heart failure based on metabolomics and zebrafish model. Front Pharmacol. (2022) 13:909084. doi: 10.3389/fphar.2022.909084, 36313322 PMC9614665

[69] Zheng C Shan L Tong P Efferth T. Cardiotoxicity and Cardioprotection by artesunate in larval zebrafish. Dose Response. (2020) 18:1559325819897180. doi: 10.1177/1559325819897180, 31975974 PMC6958657

[70] Tokunaga S Woodin BR Stegeman JJ. Plant lignan secoisolariciresinol suppresses pericardial edema caused by dioxin-like compounds in developing zebrafish: implications for suppression of morphological abnormalities. Food Chem Toxicol. (2016) 96:160–6. doi: 10.1016/j.fct.2016.07.012, 27427306

[71] Shimizu N Shiraishi H Hanada T. Zebrafish as a useful model system for human liver disease. Cells. (2023) 12:246. doi: 10.3390/cells12182246, 37759472 PMC10526867

[72] Tao X-Y Wu Y-X Li X Li F-T Dai Y-L Zheng F . Investigation of the protection from liver injury and pharmacokinetics of baijiu with fermented ginseng in rat and zebrafish models. Chin J Anal Chem. (2022) 50:100068. doi: 10.1016/j.cjac.2022.100068

[73] Gao T Lin L Yang Q Zhu Z Wang S Xie T . The raw and vinegar-processed *Curcuma phaeocaulis* Val. Ameliorate TAA-induced zebrafish liver injury by inhibiting TLR4/MyD88/NF-kappaB signaling pathway. J Ethnopharmacol. (2024) 319:117246. doi: 10.1016/j.jep.2023.117246 37778523

[74] Xiong G Deng Y Cao Z Liao X Zhang J Lu H. The hepatoprotective effects of Salvia plebeia R. Br. Extract in zebrafish (*Danio rerio*). Fish Shellfish Immunol. (2019) 95:399–410. doi: 10.1016/j.fsi.2019.10.040, 31654769

[75] Liu Y Guo J Zhang J Deng Y Xiong G Fu J . Chlorogenic acid alleviates thioacetamide-induced toxicity and promotes liver development in zebrafish (*Danio rerio*) through the Wnt signaling pathway. Aquat Toxicol. (2022) 242:106039. doi: 10.1016/j.aquatox.2021.106039, 34856462

[76] Deng D Zhao B Yang H Wang S Geng Z Zhou J . Investigating the effect and potential mechanism of rhamnetin 3-O-α-Rhamnoside on acute liver injury *in vivo* and in vitro. Pharmaceuticals (Basel). (2025) 18:116. doi: 10.3390/ph18010116, 39861177 PMC11769157

[77] Jiang M Li Z Qin X Chen L Zhu G. Regulatory role of flavonoid baicalin from Scutellaria baicalensis on AMPK: a review. Am J Chin Med. (2025) 53:771–801. doi: 10.1142/S0192415X25500296, 40374371

[78] Zhang J Deng Y Cheng B Huang Y Meng Y Zhong K . Protective effects and molecular mechanisms of baicalein on thioacetamide-induced toxicity in zebrafish larvae. Chemosphere. (2020) 256:127038. doi: 10.1016/j.chemosphere.2020.127038, 32470728

[79] Feng XH Xu HY Wang JY Duan S Wang YC Ma CM. *In vivo* hepatoprotective activity and the underlying mechanism of chebulinic acid from *Terminalia chebula* fruit. Phytomedicine. (2021) 83:153479. doi: 10.1016/j.phymed.2021.153479, 33561764

[80] Verma SK Nandi A Sinha A Patel P Mohanty S Jha E . The posterity of zebrafish in paradigm of in vivo molecular toxicological profiling. Biomed Pharmacother. (2024) 171:116160. doi: 10.1016/j.biopha.2024.116160, 38237351

[81] Meng X Yang L Liao Z Sun F Su M Mei Z. Modeling central nervous system disorders in zebrafish: novel insights into pathophysiology and therapeutic discovery. Neurobiol Dis. (2025) 216:107123. doi: 10.1016/j.nbd.2025.107123, 41015094

[82] Zhao J Yang J Yang F Wang Y Xia Q Ren F . An integrated zebrafish model and network pharmacology to investigate the mechanism of Chrysanthemum for treating metabolic dysfunction-associated fatty liver disease. Food Chem. (2025) 473:143134. doi: 10.1016/j.foodchem.2025.143134, 39893923

[83] Abu-Siniyeh A Al-Zyoud W. Highlights on selected microscopy techniques to study zebrafish developmental biology. Lab Animal Res. (2020) 36:12. doi: 10.1186/s42826-020-00044-2, 32346532 PMC7178987

[84] Burgess JL Wyant WA Abdo Abujamra B Kirsner RS Jozic I. Diabetic wound-healing science. Medicina. (2021) 57:1072. doi: 10.3390/medicina57101072, 34684109 PMC8539411

[85] Bohaud C Johansen MD Jorgensen C Ipseiz N Kremer L Djouad F. The role of macrophages during zebrafish injury and tissue regeneration under. Front Immunol. (2021) 12:707824. doi: 10.3389/fimmu.2021.707824, 34367168 PMC8334857

[86] Ding F Yu Y Zhang Y Wei S Han JH Li Z. Harnessing nutrients and natural products for sustainable drug development. Front Pharmacol. (2025) 16:1579266. doi: 10.3389/fphar.2025.1579266, 40356992 PMC12066681

[87] Garcia GR Noyes PD Tanguay RL. Advancements in zebrafish applications for 21st century toxicology. Pharmacol Ther. (2017) 161:11–21. doi: 10.1016/j.pharmthera.2016.03.009, 27016469 PMC4851906

[88] Guan F Wang R Yi Z Luo P Liu W Xie Y . Tissue macrophages: origin, heterogenity, biological functions, diseases and therapeutic targets. Signal Transduct Target Ther. (2025) 10:93. doi: 10.1038/s41392-025-02124-y, 40055311 PMC11889221

[89] Liu Y-Y Wu J-Q Fan R-Y He Z-H Li C-Y He M-F. Isoliquiritin promote angiogenesis by recruiting macrophages to improve the healing of zebrafish wounds. Fish Shellfish Immunol. (2020) 100:238–45. doi: 10.1016/j.fsi.2020.02.071, 32135341

[90] Nunan R Harding KG Martin P. Clinical challenges of chronic wounds: searching for an optimal animal model to – disease models & mechanisms. Dis Model Mech. (2014) 7:1205–13. doi: 10.1242/dmm.016782. 25359790 PMC4213725

[91] Lee S-H Ko C-I Jee Y Jeong Y Kim M Kim J-S . Anti-inflammatory effect of fucoidan extracted from Ecklonia cava in zebrafish model. Carbohydr Polym. (2013) 92:84–9. doi: 10.1016/j.carbpol.2012.09.066 23218269

[92] Chang C-C Lu Y-C Wang C-C Ko T-L Chen J-R Wang W . Antrodia cinnamomea extraction waste supplementation promotes thermal stress tolerance and tissue regeneration ability of zebrafish. Molecules. (2020) 25:4213. doi: 10.3390/molecules25184213 32937928 PMC7571120

[93] Edirisinghe S Rajapaksha D Nikapitiya C Oh C Lee K-A Kang D-H . Spirulina maxima derived marine pectin promotes the in vitro and in vivo regeneration and wound healing in zebrafish. Fish Shellfish Immunol. (2020) 107:414–25. doi: 10.1016/j.fsi.2020.10.008 33038507

[94] Santos MDF Carneiro WF Virote BDCR Silva KCDD Castro TFD Coli AP . Evaluating the bioactivity and toxicity of Siparuna guianensis Aublet (Siparunaceae) leaf extracts in zebrafish. Adv Tradit Med. (2024) 24:569–82. doi: 10.1007/s13596-023-00722-1

[95] Xu S Zhang H Li C-Z Lai P-S Wang G Chan YS . Cannabidiol promotes fin regeneration and reduces apoptosis in zebrafish embryos. J Funct Foods. (2021) 86:104694. doi: 10.1016/j.jff.2021.104694

[96] Silva KCDD Carneiro WF Virote BDCR Santos MDF Oliveira JPLD Castro TFD . Evaluation of the anti-inflammatory and antioxidant potential of *Cymbopogon citratus* essential oil in zebrafish. Animals. (2024) 14:581. doi: 10.3390/ani14040581 38396549 PMC10886050

[97] Liu W Yu H Gurbazar D Rinchindorj D Kang W Qi C . Anti-inflammatory effects and beneficial effects of the feed additive Urtica cannabina L. in zebrafish. PLoS One. (2024) 19:e0307269. doi: 10.1371/journal.pone.0307269 39018284 PMC11253947

[98] Pang S Gao Y Wang F Wang Y Cao M Zhang W . Toxicity of silver nanoparticles on wound healing: a case study of zebrafish fin regeneration model. Sci Total Environ. (2020) 717:137178. doi: 10.1016/j.scitotenv.2020.137178 32062274

[99] Manjunatha B Sreevidya B Lee SJ Liu K-C. Developmental toxicity of aristolochic acid in developing zebrafish (*Danio rerio*) embryos: cardiovascular failure and inhibited caudal fin regeneration. J Microbiol Biotechnol Food Sci. 10:e31.

[100] Cao Z Guo C Chen G Liu J Ni H Liu F . Shikonin inhibits fin regeneration in zebrafish larvae. Cells. (2022) 11:3187. doi: 10.3390/cells11203187 36291055 PMC9601185

[101] Gence L Fernezelian D Bringart M Veeren B Christophe A Brion F . Hypericum lanceolatum Lam. Medicinal plant: potential toxicity and therapeutic effects based on a zebrafish model. Front Pharmacol. (2022) 13:832928. doi: 10.3389/fphar.2022.832928 35359845 PMC8963451

[102] Rojo L Villano C Joseph G Schmidt B Shulaev V Shuman J . Wound-healing properties of nut oil from Pouteria lucuma. J Cosmet Dermatol. (2010) 9:185–95. 20883291 10.1111/j.1473-2165.2010.00509.x PMC4097019

[103] Cheng C-C Chou C-Y Chang Y-C Wang H-W Wen C-C Chen Y-H. Protective role of comfrey leave extracts on UV-induced zebrafish fin damage. J Toxicol Pathol. (2014) 27:115–21. doi: 10.1293/tox.2013-0053 25352712 PMC4110935

[104] Ho T-J Tsai P-H Hsieh C-H Lin J-H Lin Y-W Wu J-R . Role of herbal extracts of catechu from *Uncaria gambir* in the treatment of chronic diabetic wounds. Pharmaceuticals. (2022) 16:66. doi: 10.3390/ph16010066 36678562 PMC9863412

[105] Hu G Mahady GB Li S Hoi MPM Wang Y-H Lee SMY. Polysaccharides from astragali radix restore chemical-induced blood vessel loss in zebrafish. Vascular cell. (2012) 4:2. doi: 10.1186/2045-824X-4-2 22357377 PMC3316134

[106] Lam HW Lin HC Lao SC Gao JL Hong SJ Leong CW . The angiogenic effects of Angelica sinensis extract on HUVEC in vitro and zebrafish in vivo. J Cell Biochem. (2008) 103:195–211. doi: 10.1002/jcb.21403 17497682

[107] He Y-L Shi J-Y Peng C Hu L-J Liu J Zhou Q-M . Angiogenic effect of motherwort (*Leonurus japonicus*) alkaloids and toxicity of motherwort essential oil on zebrafish embryos. Fitoterapia. (2018) 128:36–42. doi: 10.1016/j.fitote.2018.05.002 29729400

[108] Zhou X Siu W-S Fung C-H Cheng L Wong C-W Zhang C . Pro-angiogenic effects of Carthami Flos whole extract in human microvascular endothelial cells in vitro and in zebrafish in vivo. Phytomedicine. (2014) 21:1256–63. doi: 10.1016/j.phymed.2014.06.010 25172787

[109] Lin S Ma H Zhang S Fan W Shen C Chen J . The combination of paeonol, diosmetin-7-O-β-D-glucopyranoside, and 5-hydroxymethylfurfural from Trichosanthis pericarpium alleviates arachidonic acid-induced thrombosis in a zebrafish model. Front Pharmacol. (2024) 15:1332468. doi: 10.3389/fphar.2024.1332468 38487165 PMC10937350

[110] Weiyang S Chienwei F Chungchih T Hanchun H Zhicheng C Hsinpai L . Therapeutic effect of Guijiajiao (Colla Carapacis et Plastri) on bone regeneration in rats and zebrafish. J Tradit Chin Med. (2018) 38:197–210. doi: 10.1016/j.jtcm.2018.04.006 32186059

[111] Zhou X Siu W-S Fung C-H Cheng L Wong C-W Zhang C . Pro-angiogenic effects of Carthami Flos whole extract in human microvascular endothelial cells in vitro and in zebrafish in vivo. Phytomedicine. (2014) 21:1256–63. doi: 10.1016/j.phymed.2014.06.010 25172787

[112] Zhang P Cao J Liang X Su Z Zhang B Wang Z . Lian-Mei-Yin formula alleviates diet-induced hepatic steatosis by suppressing Yap1/FOXM1 pathway-dependent lipid synthesis. Acta Biochim Biophys Sin. (2024) 56:621. doi: 10.3724/abbs.2024025 38516704 PMC11090856

[113] Ahmad O Wang B Ma K Deng Y Li M Yang L . Lipid modulating anti-oxidant stress activity of gastrodin on nonalcoholic fatty liver disease larval zebrafish model. Int J Mol Sci. (2019) 20:1984. doi: 10.3390/ijms20081984 31018538 PMC6515101

[114] Sa R Feng C Bai H Yin X Song L Hu X . Inhibitory effects of Mongolian medicine Yihe-tang on continuous darkness induced liver steatosis in zebrafish. Evidence-Based Complementary Alternative Medicine. (2022) 2022:5794655. doi: 10.1155/2022/5794655 35646144 PMC9142287

[115] Zhang F Zhang X Gu Y Wang M Guo S Liu J . Hepatoprotection of Lycii Fructus polysaccharide against oxidative stress in hepatocytes and larval zebrafish. Oxidative medicine cellular longevity. (2021) 2021:3923625. doi: 10.1155/2021/3923625 33680282 PMC7906805

[116] Li F Song G Wang X Sun Y Zhou S Zhang Y . Evidence for Adverse Effects on Liver Development and Regeneration in Zebrafish by Decabromodiphenyl Ethane. Environmental Science & Technology. (2023) 57:19419–29. doi: 10.1021/acs.est.3c06747 37946494

[117] Balkrishna A Lochab S Varshney A. Livogrit, a herbal formulation of *Boerhavia diffusa*, Phyllanthus niruri and *Solanum nigrum* reverses the thioacetamide induced hepatocellular toxicity in zebrafish model. Toxicol Rep. (2022) 9:1056–64. doi: 10.1016/j.toxrep.2022.03.053 35571233 PMC9097504

[118] Wang L-W Cui X-Y He J-F Duan S Liu C-R Shan C-B . Hydroxysafflor yellows alleviate thrombosis and acetaminophen-induced toxicity in vivo by enhancing blood circulation and poison excretion. Phytomedicine. (2021) 87:153579. doi: 10.1016/j.phymed.2021.153579 33991865

[119] Gong L Zhou H Wang C He L Guo C Peng C . Hepatoprotective effect of forsythiaside a against acetaminophen-induced liver injury in zebrafish: coupling network pharmacology with biochemical pharmacology. J Ethnopharmacol. (2021) 271:113890. doi: 10.1016/j.jep.2021.113890 33516931

[120] Ni B Liu Y Gao X Cai M Fu J Yin X . Isoliquiritigenin attenuates emodin-induced hepatotoxicity in vivo and in vitro through Nrf2 pathway. Comparative Biochemistry and Physiology Part C: Toxicology & Pharmacology. (2022) 261:109430. doi: 10.1016/j.cbpc.2022.109430 35944824

[121] Wang X Zhao J Zhang R Liu X Ma C Cao G . Protective effect of Hedyotis diffusa Willd. Ethanol extract on isoniazid-induced liver injury in the zebrafish model. *Drug design, development*. Therapy. (2023):1995–2015. doi: 10.2147/DDDT.S358498 PMC9249440 35783199

[122] Fan Z Liang C Zhang J Li Y Tan L Deng H . Multimodal synergistic strategies for diabetic wound healing using glucose oxidase nanocomposites: therapeutic mechanisms and nanomaterial design. Int J Nanomedicine. (2025) 20:5727–62. doi: 10.2147/IJN.S515057 40337147 PMC12056316

[123] Nachtrab G Czerwinski M Poss KD. Sexually dimorphic fin regeneration in zebrafish controlled by androgen/GSK3 Signaling. Curr Biol. (2011) 21:1912–7. doi: 10.1016/j.cub.2011.09.050 22079110 PMC3236601

